# Chromogranin B (CHGB) is dimorphic and responsible for dominant anion channels delivered to cell surface via regulated secretion

**DOI:** 10.3389/fnmol.2023.1205516

**Published:** 2023-06-26

**Authors:** Gaya P. Yadav, Haiyuan Wang, Joke Ouwendijk, Stephen Cross, Qiaochu Wang, Feng Qin, Paul Verkade, Michael X. Zhu, Qiu-Xing Jiang

**Affiliations:** ^1^Departments of Microbiology and Cell Science and of Medicinal Chemistry, University of Florida, Gainesville, FL, United States; ^2^Departments of Physiology and Biophysics, State University of New York at Buffalo, Buffalo, NY, United States; ^3^Laboratory of Molecular Physiology and Biophysics, Hauptman-Woodward Medical Research Institute, Buffalo, NY, United States; ^4^Department of Integrative Biology and Pharmacology, McGovern Medical School, The University of Texas Health Science Center at Houston, Houston, TX, United States; ^5^School of Biochemistry, University of Bristol, Bristol, United Kingdom; ^6^Wolfson Bioimaging facility, University of Bristol, Bristol, United Kingdom; ^7^Cryo-EM Center, Laoshan Laboratory, Qingdao, Shandong, China

**Keywords:** regulated secretory pathways, cell surface anion channels, dimorphic CHGB, secretory granule exocytosis, cryo-EM structures

## Abstract

Regulated secretion is conserved in all eukaryotes. In vertebrates granin family proteins function in all key steps of regulated secretion. Phase separation and amyloid-based storage of proteins and small molecules in secretory granules require ion homeostasis to maintain their steady states, and thus need ion conductances in granule membranes. But granular ion channels are still elusive. Here we show that granule exocytosis in neuroendocrine cells delivers to cell surface dominant anion channels, to which chromogranin B (CHGB) is critical. Biochemical fractionation shows that native CHGB distributes nearly equally in soluble and membrane-bound forms, and both reconstitute highly selective anion channels in membrane. Confocal imaging resolves granular membrane components including proton pumps and CHGB in puncta on the cell surface after stimulated exocytosis. High pressure freezing immuno-EM reveals a major fraction of CHGB at granule membranes in rat pancreatic β-cells. A cryo-EM structure of bCHGB dimer of a nominal 3.5 Å resolution delineates a central pore with end openings, physically sufficient for membrane-spanning and large single channel conductance. Together our data support that CHGB-containing (CHGB+) channels are characteristic of regulated secretion, and function in granule ion homeostasis near the plasma membrane or possibly in other intracellular processes.

## Highlights

- CHGB is required for appearance of robust anion conductances on the surface of neuroendocrine cells after regulated secretion.
- Native CHGB proteins exist in both soluble and membrane-bound forms in bovine pancreas and rat pancreatic β-cells and are hence dimorphic.
- Both soluble and membrane-bound forms of native bovine CHGB of nearly 100% purity reconstitute anion channels in membranes. The channels display the same high anion selectivity and sensitivity to Cl^−^ and DIDS as those made of recombinant murine CHGB.
- A near-atomic resolution cryo-EM structure of bovine CHGB dimer reveals a central cavity suitable to traverse two membrane leaflets and form a large-conductance ion channel, concordant with properties of the anion channels delivered via regulated secretion.

## Introduction

Eukaryotes rely on regulated secretion to achieve local or remote regulations in a multicellular body or in a commune of unicellular organisms (Bartolomucci et al., 2011). In vertebrates, exocrine and endocrine cells, neurons, stem cells, etc. all utilize regulated secretion to achieve physiological homeostasis (Dunzendorfer et al., 2002; Paco et al., 2009; Hur et al., 2010; Kumar et al., 2015; Shawe-Taylor et al., 2017). Regulated secretion differs from synaptic vesicle release in neurons or degranulation in immune granulocytes because of differences in physiological engagements and intracellular organelles involved (Jahn and Sudhof, 1994; Gwalani and Orange, 2018; Lettau et al., 2019; Vincent et al., 2021). Distinct from synaptic vesicles and the so-called lysosome-related organelles (LRO; also called dense core granules) in granulocytes (Mizuno et al., 2007), secretory granules are the specialized organelles for regulated secretion. There are three main intracellular steps of regulated secretion (Bartolomucci et al., 2011) – (1) granule biogenesis at the *trans*-Golgi network (TGN) to produce nascent immature secretory granules (ISGs); (2) maturation of the ISGs into dense-core secretory granules (DCSGs) that is accompanied by luminal acidification and liquid–liquid phase separation of granin proteins, cargo molecules and other components and/or amyloid-based storage of small proteins (Maji et al., 2009; Delevoye et al., 2019; Lin et al., 2022; Parchure et al., 2022); and (3) release of DCSGs via membrane fusion that is triggered by specific secretagogues from the outside. Similar to synaptic vesicle release, components of the granular membranes may stay together on the cell surface for a short while before being endocytosed and recycled back to TGN or ISGs (Pimplikar and Huttner, 1992; Willig et al., 2006). Although different granules may secrete distinct proteins or small compound molecules, they share major protein machineries for the three key steps (Bartolomucci et al., 2011). Many questions remain open on mechanistic controls of these steps (Jiang and Yadav, 2022), such as protein phase separation for granule biogenesis (Prasad et al., 2008; Maji et al., 2009; Bearrows et al., 2019; Lin et al., 2022; Parchure et al., 2022), control of granule numbers and sizes (Lin et al., 2022), homotypic fusion of ISGs (Du et al., 2016), variation in maturation rates of ISGs and aging speeds of DCSGs (Sobota et al., 2006), functional amyloid-based cargo compaction in DCSGs (Maji et al., 2009), post-release recycling of granular components (Delevoye et al., 2019), etc.

Granin family proteins are named after their organellar origin (Bartolomucci et al., 2011). They were proposed to participate in all three steps of regulated secretion (Steiner et al., 1989; Lee et al., 1999; Baumann and Saltiel, 2001; Saltiel, 2001). Secretogranin III (SgIII, also called chromogranin D [CHGD or CgD]) was reported to bind cholesterol and cargo molecules for granule biogenesis (Takeuchi and Hosaka, 2008). Secretogranin II (SgII, also called chromogranin C [CgC or CHGC]) was found to undergo liquid–liquid phase separation *in vitro* and likely contribute to the regulation of granule sizes (Takeuchi and Hosaka, 2008; Lin et al., 2022). Chromogranins A & B (CHGA & CHGB), used to be called CgA and CgB (SgI), respectively, were proposed to participate in protein aggregation-induced membrane budding in TGN (Tooze, 1998; Hordejuk et al., 2006; Bearrows et al., 2019). Their roles in biogenesis are nonessential because of strong compensatory effects in *Chga/Chgb* double knockout mice (Dominguez et al., 2018). The granin proteins may actively facilitate cargo sorting and maturation inside ISGs or release control of DCSGs (Prasad et al., 2008; Takeuchi and Hosaka, 2008; Lin et al., 2022). In normal secretory cells, CHGA and CHGB are often the most abundant among all granin family proteins. They have low sequence homology and may interact with each other and other partners (Choe et al., 2004; Hur et al., 2010; Bartolomucci et al., 2011). For CHGB, although prior studies have focused almost exclusively on its heat-stable soluble fractions (e.g., Benedum et al., 1986, 1987), partially purified native CHGB was found to bind to phospholipid vesicles *in vitro* (Yoo, 1995), and a “tightly membrane-associated form” of CHGB was detected on the surface of PC-12 cells after stimulated granule release and was attributed to a fraction of full-length protein resistant to membrane dissociation under harsh conditions except detergents (Pimplikar and Huttner, 1992). All these indicate possible presence of a membrane-inserted state (at least partially) of CHGB in addition to the soluble forms, suggesting that native CHGB might be dimorphic and its membrane-bound forms might function in both granular and plasma membranes.

Granule membranes may contain both anion and cation conductances (either channels or transporters). Liquid–liquid phase separation of cargos in DCSGs, as indicated by phase separation of chromogranins and functional amyloids of various peptide hormones (Maji et al., 2009; Lin et al., 2022; Parchure et al., 2022), leads to dense protein compaction in DCSGs. Presumably, such a phase separation is ultimately driven by vacuolar ATPase-catalyzed H^+^ translocation, but after granule release, it disappears (or is reversed) when granules face the extracellular milieu. The granular phase separation thus requires proper ionic homeostasis, pH regulation and osmolality balance across granular membranes (Jiang and Yadav, 2022). Permeation of granular membranes to proton and Cl^−^ is known (Johnson and Scarpa, 1976; Thevenod, 2002), but genetic identity of the Cl^−^ conductors remains unknown (Nobile et al., 2000; Kelly et al., 2005; Hordejuk et al., 2006; Stolting et al., 2014; Comini et al., 2022).

Our recent finding of a large-conductance high-selectivity Cl^−^ channel made of recombinant murine (m) CHGB of ~100% purity suggests a possible connection of the membrane-bound CHGB to the Cl^−^ conductances in granular membranes (Yadav et al., 2018). The activities of recombinant mCHGB were recorded from billions of channels in vesicles by Ag/AgCl electrodes and Cl^−^ flux assays as well as from a few to dozens of channels in planar lipid bilayers (Yadav et al., 2018). With both negative and positive controls and exquisite protein purity, the likelihood of any unknown contaminants to generate such functional data was estimated at <10^−7^ (Jiang and Yadav, 2022). Even though a high-resolution structural model is still needed to reveal the transmembrane topology of the CHGB channel, physical laws for diffusion-limited ion conduction dictate that the channel must cross a lipid bilayer fully and contain a central pore suitable for ultra-fast ion crossing (~10^7^ – 10^8^ per second) (Hille, 2001). Although sequence analysis and structure prediction of CHGB suggest that its monomer is not well ordered, CHGB oligomers can form a channel, reminiscent of many well-studied dimorphic proteins capable of switching from the soluble to membrane-integrated state in a lipid-dependent manner (Parker and Feil, 2005; Johnstone et al., 2021). Our prior data suggested that each CHGB monomer contains two or more amphipathic helices and four monomers together provide structural elements to enclose likely two hydrophilic pores for anion selection and ultrafast conduction (Yadav et al., 2018). On the other hand, native CHGB in neuroendocrine cells contains dozens of posttranslational modifications (PTMs) that might be cell-specific (Rosa et al., 1985; Conlon et al., 1992). This raises the question on whether the native CHGB can still function as an anion channel on the cell surface after granule release and can fulfill important functions there. In this study, we addressed these questions by analyzing the regulated secretion-induced anion channels on the cell surface and the key properties of native CHGB in contributing to such channels. In a companion paper (Yadav, #2), we will address potential intragranular functions of the CHGB-mediated channels in regulated secretion.

## Materials and methods

### Molecular cloning of CHGB and its different mutants in pFastbac1

Preparation of all constructs followed the same general procedure as described before ^41^ unless separately described.

### Knockdown of CHGB in neuroendocrine cells

INS-1 (832/13) cells of 40% confluency were seeded on the day before transfection. Cells were transfected with RNAiMAX (Life Technologies) that was mixed with varying amounts of siGENOME non-specific siRNAs (D-001210-01-05; CTL siRNAs or scRNAs) which were scrambled from the CHGB-specific sequences, or CHGB-targeting siRNA-SMART pool (M-099320-01-0005; CHGB siRNAs; or the 2^nd^ and/or 4^th^ siRNA) from Dharmacon. Forty-eight hours after transfection, the cells were changed into a fresh medium. For evaluating off-target effects, individual siRNAs from the mixture were tested and compared with each other and with the mixture. For CHGB, the 2nd and 4th siRNAs were more effective than the 1st and 3rd ones. The mixture of 2nd and 4th siRNAs was equally effective. Because the different combinations were found to have the same effects, we concluded that the siRNA knockdown of CHGB was highly specific. Testing with siRNAs targeting CLC-3 and CLC-5 further strengthened this point. Knockdown of CLC-3, CLC-5, ANO-1 and ANO-2 followed the same protocol. For each of them, a dose-dependent response was measured with the same number of cells and was used to guide the selection of a minimal concentration of siRNAs that reached suppression of 90% or more protein expression. Often 75–100 nM siRNAs were used.

To examine the effectiveness of the siRNA knockdown, we lysed the same number of cells for western blotting. 96 h after transfection, cells were lifted by incubation with 10 mM EDTA in 1 x PBS. After incubation for a couple of minutes, the cells were resuspended and washed twice with PBS in order to remove extracellular EDTA. The cells were subsequently changed into a lysis buffer containing 50 mM Tris, pH8.0, 10 mM NaCl, 1.0 mM EDTA and 1.0 mM DTT, and went through three freeze–thaw cycles between liquid N_2_ temperature and 37°C (Zhang et al., 2014). Afterwards, cell lysates were incubated in ice for 30 min and then centrifuged at 18,000× *g* for 30 min to remove cell debris. Concentration of the released proteins was measured using a Nano-Drop 2000c spectrophotometer (Thermo Scientific) or more accurately using the Bradford method (Bio-Rad). ~ 20 μg of proteins from cell lysates were loaded into each lane in an SDS-PAGE gel. Western blotting for CHGB was done with an anti-CHGB antibody (goat-anti-mouse antibody; catalog #: sc-1,489 from Santa Cruz Biotechnology, Santa Cruz, CA, USA). An HRP–conjugated donkey-anti-goat 2nd antibody (Santa Cruz) was used for detection using the Super Signal West Pico chemiluminescent substrate (Thermo Scientific).

### Whole-cell patch clamp recordings from PC-12 cells

PC-12 cells were cultured in high-glucose DMEM supplemented with 10% heat-inactivated fetal bovine serum (FBS) at 37°C inside a 5% CO_2_ incubator. Transfection with siRNAs was performed as described above. Cells were changed from the transfection mixture into the regular culture medium after incubation for 8 h.

Transfected cells were cultured for 24–48 h before electrophysiological recordings. The cells were seeded on 12-mm coverslips for 3–4 h before whole-cell recordings. A recording pipette was pulled from a borosilicate glass tubing with a thin filament inside (Sutter Instrument, Novato, CA, USA). The electrodes always had a serial resistance of 5–8 MΩ when filled with a regular intracellular solution containing (in mM) 155 K-gluconate, 10 EGTA, 1.0 CaCl_2_, 2.0 MgCl_2_, 2.0 Mg-ATP, 5.0 HEPES, pH 7.2. A normal bath solution contained (in mM) 140 NaCl, 4.8 KCl, 2.5 CaCl_2_, 1.2 MgCl_2_, 5.0 glucose, 20 HEPES, pH 7.4. A high K^+^ bath solution contained (in mM) 104.8 NaCl, 40 KCl, 2.5 CaCl_2_, 1.2 MgCl_2_, 5.0 glucose, 20 HEPES, pH 7.4. A Cl^−^-free bath solution contained (in mM) 140 Na-gluconate, 4.8 K-gluconate, 5.0 glucose, 20 HEPES, pH 7.4, whose free Ca^2+^ is ~1 μM. The NMDG-Cl solution contained (in mM): 155 NMDG-Cl and 20 HEPES (pH7.4). Cells were continuously perfused with the bath solution through a gravity-driven multi-outlet device with the desired outlet placed about 50 μm away from the cell being recorded. Only one cell was recorded from each coverslip.

Individual cells were voltage-clamped in the whole-cell mode using an EPC9 amplifier (HEKA Instruments, Bellmore, NY, USA). Voltage commands were delivered from the PatchMaster program (version 2×90.1; HEKA). Currents were recorded at 10 kHz. Cells were held at either −80 or 0 mV. Voltage pulses were applied either every second or ever 2 s.

### Western blotting of vATPase subunit ATP6V0A2.

Cells were treated the same as above before they were lysed by three freeze–thaw cycles. Roughly 20 μg of lysate protein was loaded in each lane, separated by SDS-PAGE, and detected by western blotting with an anti-ATP6V0A2 antibody (Abcam).

### Purification of native bCHGB from bovine pancreatic granules and measurements of channel activity by flux assays and recordings from lipid bilayers

The protocol was modified from a published procedure ^48^. Bovine pancreas was collected from the slaughterhouse at the UF School of Veterinary Sciences, cut into smaller pieces, immediately flash-frozen in 1 x PBS plus 1.0 mM PMSF and stored at −80°C until being used for protein purification. The frozen pancreatic tissue was thawed on ice and cut into small cubic pieces (0.5–1.0 cm) with a pair of sterilized scissors in the isolation buffer (20 mM MOPS-KOH, 0.34 M sucrose, 5.0 mM EDTA, pH 6.5) supplemented with a cocktail of protease inhibitors and 1.0 mM PMSF. The diced tissues were homogenized in a pre-cooled kitchen blender (15 pulses). The homogenates were centrifuged at 2,200× *g* for 10 min at 4.0°C. The supernatant was filtered through two layers of cheese cloth and the filtrates were centrifuged at 17,000× *g* for 20 min at 4.0°C. The crude fraction in the pellet was resuspended gently in the isolation buffer and layered onto a one-step density gradient of 1.6 M sucrose dissolved in the isolation buffer. After centrifugation at 60,000× *g* for 1 h at 4.0°C, different fractions from the gradient were collected and tested for the protein content. The pellet was resuspended and lysed in a buffer made of 1.0 mM MOPS-KOH pH 6.5 and protease inhibitors. After three freeze–thaw cycles between liquid nitrogen temperature and 37°C, the suspension was centrifuged at 100,000× *g* for 1 h in order to separate the soluble content (referred as the soluble fraction) in the granular cores from the granular membranes (called the membrane-bound fraction). The pellet containing membrane-associated CHGB was extracted using 2.0% NP40 for 1 h in a cold room. The supernatant contained the soluble fraction of CHGB and was also extracted with detergents before protein purification. The lysates from both fractions were applied to the HiTrap-QFF column separately and the bCHGB protein was eluted with a NaCl gradient. The fractions containing bCHGB (based on western blot) were pooled together and subjected to 3–4 rounds of size-exclusion chromatography in a Superose 6 column. The bCHGB protein was monitored at every step by western blotting. After three separate runs, the peak fractions contained bCHGB with at least 80% purity. In the biochemical preparation in detergents, the contaminating protein in the Coomassie-blue stained SDS-PAGE gels were identified as HSP protein (HSPA9, 74 KDa) by mass spectrometry. Besides the HSP protein, bCHGB accounts for nearly all of the rest protein in our biochemical preparations. Further, the HSP proteins were not associated with vesicles after reconstitution, and were removed after vesicle floatation in a Ficoll 400 density gradient. The separated vesicles contained only one visible band of bCHGB (~99.3% pure) by SDS-PAGE analysis and comparison of the digitized densities of the three band regions detected by western blotting of CHGB. These data support that after vesicle reconstitution, the CHGB is the only detectable protein in vesicle membranes. The nearly 100% purity of bCHGB is critical for assignment of the channel function to the protein in both the flux assays from channels in billions of vesicles in suspension and the bilayer recordings from a few to dozens of channels every time.

For light scattering-based flux assays and electrical recordings of bCHGB in vesicles, the full details were specified before (Yadav et al., 2018). We found that inclusion of ~5% cholesterol by weight into the lipid mixture for reconstitution enhanced CHGB channel activity. The protein/lipid weight ratio was kept at 1:25 for reconstitution, and either egg PC or POPC was used as the main lipid components. The light-scattering measurements were done in a FlouroMax®-4 system (HORIBA) in the lab. For patch clamp recordings of bCHGB channels fused in planar lipid bilayers, 150 / 30 (cis/trans) mM KCl was used as the starting solutions with a reversal potential of −41.2 mV for Cl^−^ and + 41.2 mV for K^+^ at room temperature (20 °C). 5.0 mM MES-KOH (pH5.6) was used to buffer the pH levels on both sides. Except for the studies of channel inactivation in the presence of 2.0 mM Mg^2+^ or Ca^2+^, no divalent cations were added, nor was EGTA or EDTA present as before.

### High-pressure freezing and immuno-electron microscopy (HPF immuno-EM)

Rat pancreatic islets were isolated and high-pressure frozen as described (van Weering et al., 2010; Fava et al., 2012). They were subsequently freeze-substituted to Lowicryl HM20 (van Weering et al., 2010) using the pre-prepared MonoStep version (PolySciences Inc.). After UV polymerization 70 nm sections were collected on pioloform-coated copper slot grids. Immuno gold double labeling was performed as described (Verkade et al., 1997; van Weering et al., 2010) using a mouse monoclonal antibody against insulin (Cell Signaling, I6B10) at a dilution of 1:100, and a polyclonal rabbit CHGB antibody (H-300, 1:10). The primary antibodies were detected using 6 and 12 nm gold coupled anti-mouse and anti-rabbit antibodies (Aurion, Wageningen, The Netherlands). Experiments were carried out both with anti-mouse 6 and 12 nm gold and anti-rabbit 12 and 6 nm, respectively. Controls included leaving out one or both primary antibodies which resulted in the lack of the respective gold label(s). To make a robust statistical analysis the density of the stained molecules per granule was kept low, and ~ 320 granules were included in the dataset for image analysis (next section). Samples were analyzed on a FEI Tecnai12 BioTwin TEM, equipped with a bottom-mount Ceta camera (Thermo Fisher). Images were exported as TIF files with scalebars incorporated so that they could be imported and calibrated in Fiji for subsequent analysis.

### Quantitative analysis of HPF immuno-EM data

Analysis of insulin and chromogranin B distribution inside insulin granules was performed using a Modular Image Analysis automated workflow plugin for Fiji (Schindelin et al., 2012; Rueden et al., 2017; Cross, 2018). The first step in the workflow was to enhance insulin and chromogranin B signals using an implementation of the WEKA Trainable Segmentation plugin for Fiji (Arganda-Carreras et al., 2016). The resulting probability images were then binarized using the Otsu method (Otsu, 1979) and optimized with the ImageJ hole-filling and watershed binary processes. Insulin and chromogranin B particles were characterized as contiguous regions in the binarized images. Those particles with cross-section areas smaller than 20 nm^2^ (<5 nm) were assumed to correspond to noise and were removed from further analysis. Particles with areas between 20 nm^2^ and 65 nm^2^ were classed as insulin and any particles with areas greater than or equal to 65 nm^2^ (>9 nm diameter) were classed as chromogranin B. Ellipses were fit to each identified particle using the BoneJ library (Doube et al., 2010) and any particle with eccentricity greater than 0.75 was removed from further analysis. *Ring* objects were manually identified in each image using the ImageJ freehand selection tool. Any insulin or CHGB objects falling clearly outside a *Ring* were excluded from further analysis. Finally, the distance from the centre of each particle to the edge of the containing *Ring* was measured.

### Fluorescence imaging and analysis

Fluorescence images were collected using a Zeiss LSM710 inverted confocal microscope with an oil-immersion objective (60×, 1.25 numerical aperture). Zeiss LSM800 was used in a similar way. Excitation at 458 nm and 488 nm was achieved by an argon laser. To minimize bleed-through artifacts, emission channels were chosen as follows: green channel fluorescence I_488 nm_ (488 nm excitation) was detected from 500 nm to 550 nm; cyan channel I_458 nm_ (458 nm excitation) was detected from 500 nm to 550 nm. For detectors with PMT, the PMT voltage was kept constant. Laser scanning was performed using 400 Hz line frequency, 512 × 512 pixel format and a pinhole aperture set at 1 Airy disk. To detect the surface puncta, the confocal plane was set at the cell surface using the DIC image as a guide. To minimize nonspecific labeling, cells were blocked with mouse serum before antibody labeling and BSA was added in the buffers for antibody labeling. Image processing was performed using customized ImageJ (US National Institutes of Health) macros that could be applied to sets of images in a consistent and unbiased way.

### Single particle cryo-EM analysis of CHGB

For cryo-EM studies, we followed a similar procedure as published (Yadav et al., 2018). The bCHGB proteins (dimers) purified in detergents were prepared for cryo-EM imaging in a Glacios/Falcon 4 system at HWI that was operated at 200 kV. A total electron dose used was ~56 *e_0_*/Å^2^, spread among 30 frames over a period of 2.6 s. The defocus range was −0.8 to −2.0 microns, and the calibrated pixel size was 0.526 Å/pixel at the specimen level. Due to the extensive sorting of single particle images in the past a large dataset was built in multi-day data collection sessions. After multiple-rounds of 2D-classification and interactive selections of the good particles, a dataset of ~35,000 particles were separated, and gave rise to high quality 2D class averages, which led to a converging 3D reconstruction at a nominal resolution of 6.8 Å (FSC = 0.143) in Relion 3.1 (Scheres, 2020). *Ab initio* reference models were generated in cisTEM (McSweeney et al., 2020). C2 symmetry was imposed at the end because of strong two-fold symmetry in the 2D class averages.

These initial analyses suggested that a larger dataset from a couple of weeks of data collection would be suitable to improve the resolution to 3.0–4.5 Å. We then collected nearly 90,000 movies from the same types of specimens at the Purdue University using a Titan Krios operated at 300 kV with a total dose of ~52 *e_0_*/Å^2^ over 40 frames. Data collection was done in a K3 DED without energy filter and the pixel size was calibrated to be 1.08 Å after 2×2 binning out of the super-resolution mode. The defocus range of −0.5 to −3.0 μm was used. The same general procedure was used for image analysis, except that for CHGB dimers it was important to examine the 2D class averages carefully in order to find the ones of the best quality. For proper 2D analyses in cryoSPARC (Punjani et al., 2017), the whole dataset was split into three subsets till 2D classification was done. The three subsets of chosen particles were then combined for further refinement. The *ab initio* refinement in cryoSPARC generated one reference model that resembled what was produced in cisTEM and then used for further refinement. After 3 rounds of 3D classification and refinement, the best map reached ~3.6 Å, and the other maps were all worse than 4.0 Å. We optimized the final map in Phenix by density modification, before using it for the map-to-model procedure, which found a short 3-helix bundle in the density. We then tested the sharpened cryo-EM map against DeepTracer, Model_Angelo, and I-TASSER. The model from I-TASSER was similar to what Phenix did (Moriarty et al., 2020; Pfab et al., 2021; Zhou et al., 2022; Jamali et al., 2023). The main chain residues (~1,250 residues) detected by DeepTracer and Model_Angelo agreed relatively well with each other. The one from the latter detected most short alpha-helical segments pretty well. We thus used it for modeling. As a test of the programs, we used a 3.0 Å map of murine TRPV3 in nanodisc calculated from the data collected in the same microscopic system at Purdue, for which the model from DeepTracer and Model_Angelo agreed fairly well with the model built before. These studies suggested that the resulted model should probably be trusted at the main chain level, because of high uncertainty in detecting the side chains at the nominal 3.6 Å. Because no prior information is available, except for the helical segments from sequence analysis and a disordered model by AlphaFold2 (Jumper et al., 2021), we did not go further to optimize the model, other than using it to generate a poly-Ala model. It is foreseeable that with more data and using the same strategy we will be able to improve the resolution to ~3.0 Å and enhance the confidence in side-chain recognition.

### Statistics and reproducibility

A strong version of the large number theorem is applicable to the analysis of a large number (> 45) of individual cells or granules from cells randomly selected in every repeated experiment, and analysis of pooled data from different experiments thus gained statistical significance better than treating individual sets separately. For comparison of unpaired data from two different conditions, two-sided Student’s *t*-test with proper posterior adjustments was always applied. All experiments were repeated at least 3–4 times independently for statistical comparison.

## Results

### Anion channels delivered to cell surfaces by regulated secretion require CHGB

**Figure 1:**
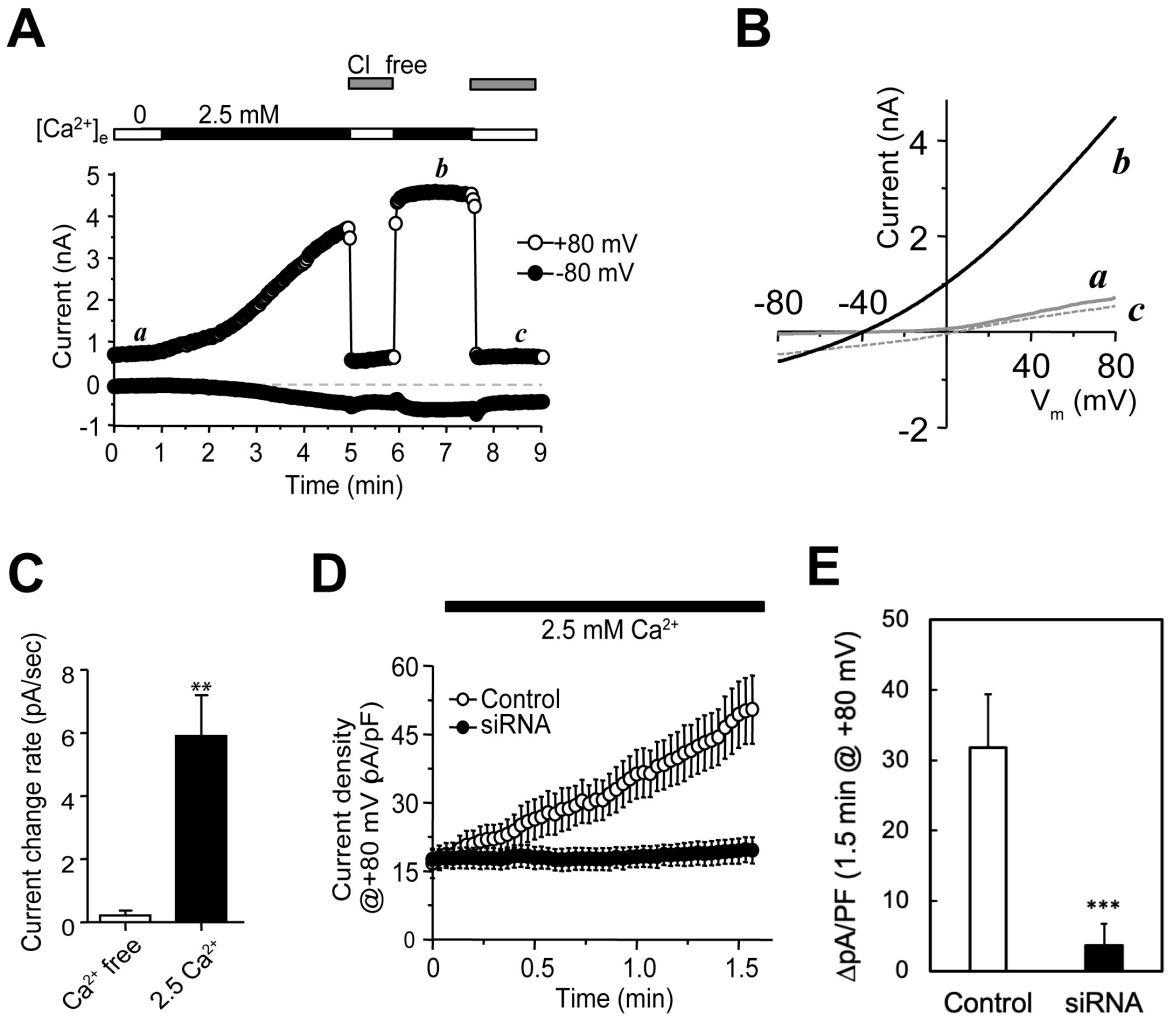
Recordings of native anion channels in plasma membranes emerging in parallel to granule release in PC-12 cells. **(A)** Whole-cell recordings from a PC-12 cell (top bars; typical of >10 cells) that was initially perfused with a Ca^2+^-free solution and then with a normal bath containing 2.5 mM Ca^2+^. The microelectrodes contained low Cl^−^. Holding potential was −80 mV. A voltage protocol made of −80 to +80 mV in 100 ms, 80 mV for 50 ms, and 0 mV for 400-ms was delivered at 1.0 Hz. Whole-cell currents were small and stable in the Ca^2+^-free bath (first period including time point *a*) and increased gradually with Ca^2+^. Switching the cell to a Cl^−^-free solution abolished the increased outward currents. After 5 min, the outward current at +80 mV became saturated (fourth period including time point *b*), which was almost completely abolished after removal of extracellular Cl^−^ (fifth period including time point *c*). The inward currents at −80 mV showed a much smaller Ca^2+^-dependent increase and were much less sensitive to removal of extracellular Cl^−^. **(B)** Current–voltage (I–V) curves elicited by voltage ramps at time points *a*, *b*, and *c*. The trace at time point *b* had weak outward rectification. Traces at time points *a* and *c* showed ~0 mV reversal potential and different rectification. **(C)** Average current change rate (mean ± *SEM*; in pA/s) at +80 mV for cells in 0 (*n* = 5) and 2.5 mM Ca^2+^ (*n* = 14) solutions. **p < 0.05. **(D)** Current development at +80 mV in cells transfected with sequence-scrambled (control; open circles) and CHGB-targeting siRNAs (siRNA; solid circles). The pipette solution contained NMDG-Cl. A voltage protocol, made of −80 to +80 mV in 500 ms, 80 mV for 50 ms and − 80 mV for 50 ms, were applied at 0.5 Hz. Cells were in Ca^2+^-free bath first and then in a normal bath with 2.5 mM Ca^2+^. Data points are mean current densities at +80 mV from 6 Control and 7 CHGB siRNA-transfected cells. **(E)** Comparison of changes in average current density over 1.5 min for the same groups of cells showed in panel **(D)**. ***p < 0.005.

We reasoned that if any anion conductances (channels or transporters) in secretory granules remain active on the cell surface after regulated secretion, they should be accessible for patch clamp recording. To test it, we performed whole-cell recordings on rat PC-12 cells, a well-studied neuroendocrine cell line containing monoamine-secretory granules (Höltje et al., 2000). At a holding potential of −80 mV, a voltage protocol consisting of a ramp from −80 to +80 mV in 100 ms, a delay at 80 mV for 50 ms, and a step to 0 mV for 400-ms was applied at 1.0 Hz to stimulate intermittent granule release and record whole-cell currents simultaneously. The depolarization steps allowed Ca^2+^ influx through voltage-gated calcium channels (VGCCs), which in turn triggered Ca^2+^-dependent exocytosis of readily releasable granules (RRGs) (Rizo and Xu, 2015). A gradual increase of currents with weak outward rectification was detected in the presence, but not absence, of 2.5 mM extracellular Ca^2+^ (Figures 1A–C, using currents at +80 mV for quantitation). The increases of outward currents were suppressed completely when extracellular Cl^−^ was replaced by gluconate (Figures 1A,B), verifying that Cl^−^ influx into the cell, instead of cation efflux from cytosol, contributed nearly all of the increase in outward currents. The remaining currents likely stemmed from either small cation conductances, weak permeation of gluconate through the anion channels or non-selective channels, which all should cause a right shift of the reversal potential from the calculated Cl^−^ Nernst potential of −82 mV. The 0 mV reversal potential of trace c (Figure 1B) suggests that the cation influx and efflux are nearly balanced at 0 mV and the contribution of nonselective ion channels is also small. The measured reversal potential of ~ −62 mV (at the crossing point of traces *b* and *c* in Figure 1B) yielded a low limit for the permeation ratio of Cl^−^ vs. Na^+^, *P_Cl_/P_Na_* > 25, if permeation of other ions is too small to consider. Under such conditions, chloride ions carried >95% of the increased currents that accompanied granule release, suggesting that cation channels in DCSG membranes are of very low abundance if existent (Thevenod et al., 2000; Hordejuk et al., 2006). After ~5 min, the current increase reached a plateau (time point *b* in Figure 1A), probably due to depletion of RRGs. The total chord conductance was increased by ~28 nS per cell, which unlikely stemmed from fS ion transporters like CLC-3 or − 5. These data suggest that granule release induces incorporation of functional anion channels to the plasma membrane, which may derive from channels that reside originally in the granule membranes. However, it was equally possible that stimulated granule release activated silent channels in cell membranes via unknown mechanisms or delivery of channels through other trafficking vesicles.

Although potential anion conductances in granules have been known for >45 years, their molecular identify remains unclear (Johnson and Scarpa, 1976; Johnson et al., 1985; Thevenod, 2002). CLC-3 was once considered (Deriy et al., 2009), but now is less favored because it works as a Cl^−^/H^+^ exchanger with a fS conductance and exhibits strict outward rectification (Guzman et al., 2013). The latter also differs from the weak rectification in Figure 1B, and makes it unable to conduct well Cl^−^ fluxes into secretory granules (inward currents) (Deriy et al., 2009). By contrast, the Cl^−^ channel reconstituted by recombinant mCHGB is mostly open at membrane potentials ranging from −60 to +60 mV and has a single channel conductance of ~140 pS in near-physiological Cl^−^ concentrations (Yadav et al., 2018), which is sufficient to underscore the currents shown in Figures 1A,B. To test this possibility, we used siRNAs to knock down CHGB expression. CHGB-specific siRNAs at 100 nM suppressed its protein expression in cells by >95% (Supplementary Figures S1A,B) whereas sequence-scrambled control (**CTL**) siRNAs produced no detectable effects. Among the four CHGB siRNAs in the mixture, each alone knocked down CHGB efficiently, with siRNAs 2 and 4 being ~2 fold more effective than the other two (Supplementary Figure S1C). In whole-cell recordings of PC-12 cells transfected with the four CHGB siRNAs, either individually or in combinations, we detected almost no depolarization-induced increase in Cl^−^ conductance, while cells transfected with the CTL siRNAs still showed robust increases (Figures 1D,E). Here, because Ca^2+^ influxes still happened robustly during exocytosis, contribution of Ca^2+^-activated chloride channels (CACCs) to the observed increase in Cl^−^ conductance, if any, should still be present in the CHGB-knockdown cells, and thus is negligibly small. In these experiments, NMDG-Cl in the pipette solution was used to suppress outward cation currents. The background level of outward currents at +80 mV seen in Figure 1B was decreased from ~800 pA (point a in Figure 1B) to ~350 pA by intracellular NMDG (Figure 1D), strengthening further that the contribution of cation channels or non-selective channels is very small. The fact that the individual CHGB siRNAs and their mixtures yielded the same results helped rule out off-target effects.

The above results demonstrated that CHGB is essential to the increased Cl^−^ conductances accompanying granule release. However, there might be several ways for the CHGB protein to contribute to such a change. First, CHGB may *directly* form the anion conducting pores that were initially in granule membranes and emerged in the plasma membrane after granule release. Second, if the conductances arose from silent channels already present in the plasma membrane or delivered by other intracellular vesicles, CHGB might either *directly* trigger their activation or be *indirectly* involved due to its role in granule exocytosis. In the latter case, CHGB knockdown could alter the availability of other factors critical to channel activation or impair the expression and/or plasma membrane trafficking of the channels.

**Figure 2:**
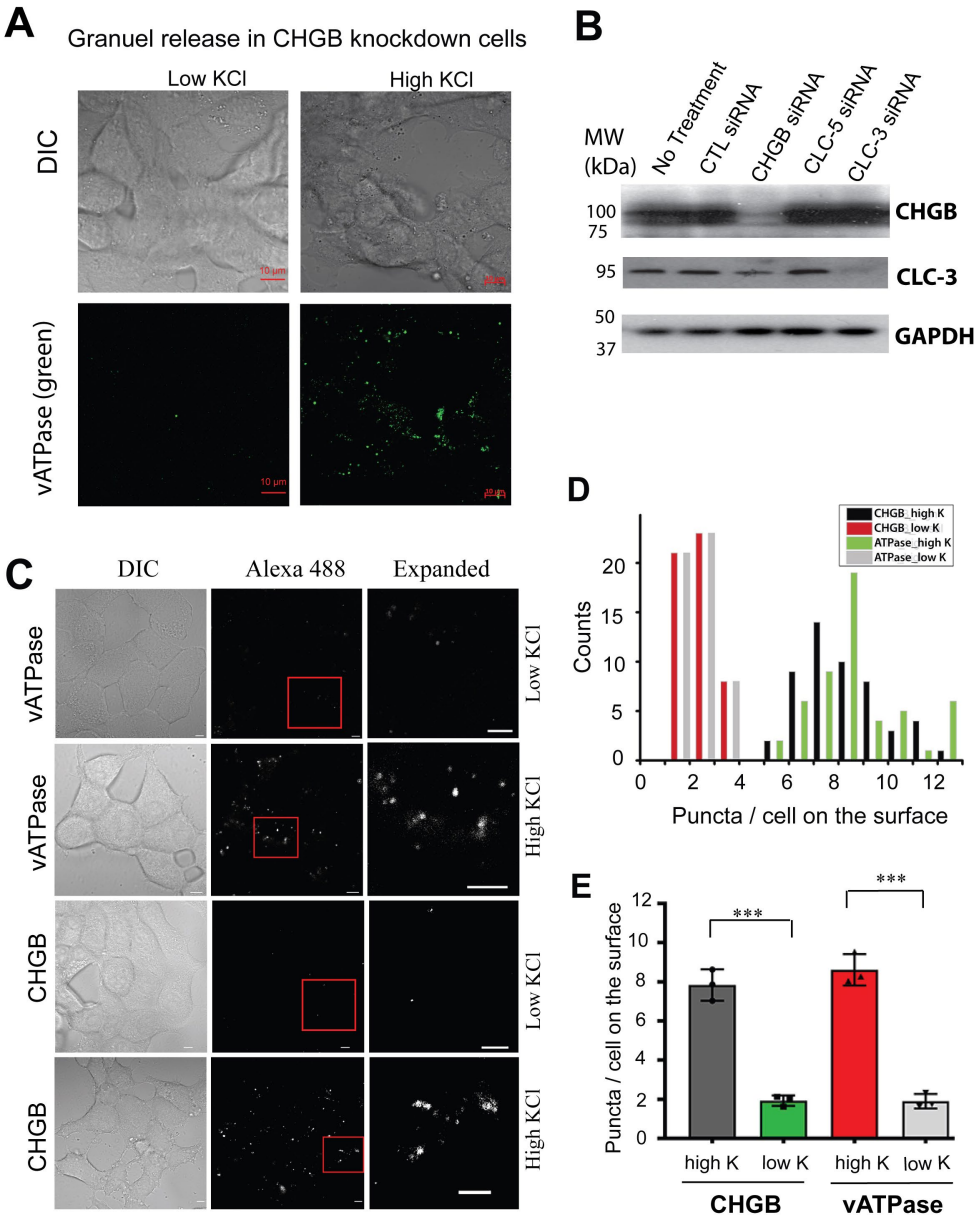
Native CHGB released as surface puncta after stimulated granule exocytosis. **(A)** In CHGB knockdown cells, vATPase (green puncta) was released to the cell surface when INS-1 cells were treated with high KCl (55 mM; right row). Cells treated with normal low KCl (4.8 mM) were showed as negative control with a very low basal level of labeling. **(B)** CHGB siRNAs and CLC-3 siRNAs showed specific knockdown of the target proteins detected by western blotting with no off-target effects. CLC-5 siRNA did not affect CHGB or CLC-3. CTL-siRNA had not effect, either. GAPDH serves as loading controls. **(C)** Normal INS-1 cells released vATPase and CHGB as surface puncta after high KCl-stimulated granule release. Low KCl treated cells were analyzed as negative control, showing a basal level of granule release. Anti-ATP6V0A2 (rows 1&2) and anti-CHGB (rows 3&4) antibodies were used to label cells on ice before an Alexa 488-conjugated secondary (2nd) antibody was introduced for confocal FM. **(D)** Numbers of CHGB or vATPase puncta on the top surfaces of the cells under different conditions (~50 cells) in panel **(C)**. **(E)** Average number of puncta per cell from 4 different experiments. ***p < 0.001. Errors*: s.d.* (*n* = 4).

To evaluate these possibilities, we first assessed whether CHGB knockdown suppresses stimulated granule release completely by detecting vacuolar H^+^-ATPase (vATPase) from the extracellular side in cells depolarized with high [KCl] (e.g., Supplementary Figure 2). CHGB knockdown did not prevent high K^+^-induced translocation of vATPase to the cell surface (Figure 2A), consistent with earlier observations that the machineries for biogenesis and release of secretory granules still function relatively well (at least 40–70% of the wild-type) in cells from *Chgb^−/−^* or *Chga/Chgb* double knockout mice because CHGB’s nonessential roles can be substituted by other proteins (Obermüller et al., 2010; Zhang et al., 2014; Dominguez et al., 2018; Bearrows et al., 2019). Second, to rule out the possibility that CHGB siRNAs acted non-specifically on other Cl^−^ channels/transporters that were proposed to underlie the granule membrane anion conductances, we measured ClC-3 (as a non-CHGB target) and CHGB protein levels in cells treated with siRNAs targeting CHGB, CLC-3, or CLC5. We detected no effects of CHGB knockdown on CLC-3 expression, and inversely, no effects of CLC3/5 knockdown on CHGB expression (Figure 2B; Supplementary Figure 1D). Together, these data support that the near-100% removal of the regulated secretion-induced anion conductances by CHGB knockdown (Figures 1D,E) did not result from defects in granule biogenesis or stimulated granule exocytosis, or mistargeting of the siRNAs on other anion channels or transporters. In addition, the fast increase of anion conductance right after each depolarization pulse happened in less than 1 s (Figure 1A), which is inconsistent with any mechanism that involves transcription of new genes or translation of new proteins regulated by CHGB because of the short time and also because CHGB is not known to be a transcription factor. Therefore, we next focused on CHGB being *directly* involved in the anion channels delivered with the granule membranes, either as the pore-forming subunits or as an auxiliary subunit physically associated with the unknown pore-forming subunit. We refer these two types as CHGB-containing or CHGB+ channels.

Because CHGB is an obligate granule protein, regulated secretion in vertebrates hence delivers CHGB+ anion channels to the cell surface. To verify this prediction, we compared numbers of surface CHGB and vATPase puncta on INS-1 cells treated with low and high concentrations of KCl (Figures 2C–E; Supplementary Figure 2). In the low KCl solution, a very small number of CHGB puncta were detected on the cell surface, resembling the small number of vATPase puncta (Figures 2C–E), indicting a low level of sporadic granule release in the absence of depolarization. However, in the high KCl solution, the numbers of surface CHGB and vATPase puncta were both increased significantly (Figures 2C–E), showing that with depolarization CHGB proteins on the cell surface are in the right place and could underlie the anion conductances observed in Figure 1.

### Native bovine CHGB is dimorphic and able to reconstitute anion channels in membranes

**Figure 3:**
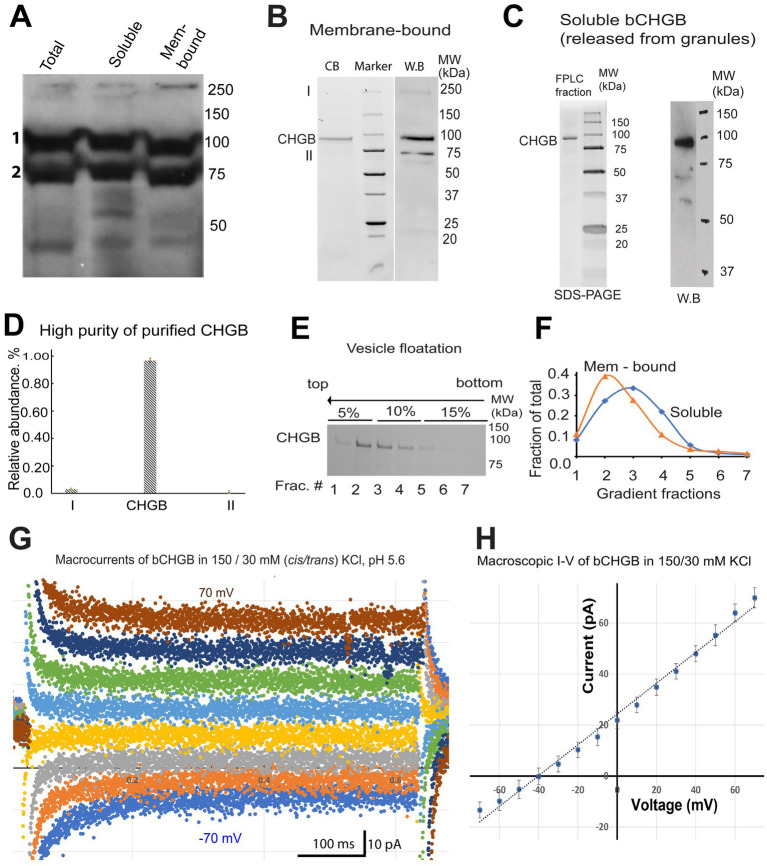
bCHGB is dimorphic and reconstitutes anion channels in membrane. **(A)** Western blotting of mature and immature bCHGB (band 1 and 2, respectively) from total granule extract, soluble and membrane-bound granule fractions. Each lane was loaded with 20 μg total proteins. **(B)** Purified bCHGB from membrane-bound granule fractions of bovine pancreatic granules. bCHGB peak fraction eluted from a Superdex 200 was analyzed by Coomassie blue-stained SDS-PAGE (CB, left lane). Western blotting of bCHGB fraction was showed on the right (WB). **(C)** Purified bCHGB extracted from the soluble granule fractions was fractionated by gel-filtration FPLC, and assayed by Coomassie blue-stained SDS-PAGE (left). W.B. was done to confirm protein identity (right). **(D)** Quantification of bCHGB and two other bands in the left lane of panel **(B)**, showing ~99.2% purity of bCHGB. **(E)** bCHGB vesicles made of DOPC: sphingomyelin: cholesterol were floated from bottom (right) to top (left) in a three-step Ficoll 400 density gradient (5, 10 and 15%), and assayed by Coomassie blue-stained SDS-PAGE. **(F)** bCHGB bands in the seven fractions from the density gradient in panel **(E)** were quantified and normalized to show the relative protein distribution (blue). Similar results of bCHGB vesicles from membrane-bound granule fractions were showed in orange. **(G)** Typical macroscopic currents from bCHGB channels in lipid bilayers with 150/30 mM KCl and 5.0 mM MES-KOH, pH 5.6. No obvious inactivation was observed in the absence of Mg^2+^ or Ca^2+^. Holding potential @ 0 mV; voltage pulses: −70 mV to +70 mV with 10 mV increments in the middle segment of 0.8 s. For clarity, only currents from 20 mV steps are presented. **(H)** I–V curve from multiple recordings as exemplified in panel **(G)** (Error bars: *s.d.*, *n* = 4). Linear fitting (dotted line) of the data points (dots) yielded a reversal potential of −40.5 mV, close to the Nernst potential of Cl^−^, −41.2 mV, and suggesting a P_Cl_/P_K_ ~ 130. The chord conductance is ~600 pS, suggesting ~5 channels in the bilayer.

Although the recombinant mCHGB formed anion channels in the absence of other proteins (Yadav et al., 2018), whether native CHGB proteins can do the same remained unclear. The abundant PTMs were likely altered or missing in the recombinant CHGB prepared from insect cells (Rosa et al., 1985; Watkinson et al., 1993). Therefore, to test whether native CHGB is a candidate of the pore-forming subunit of the delivered anion channels, we characterized bovine (b) CHGB purified from bovine pancreas, which is a good source of native CHGB proteins for biochemical preparations. We adapted two published protocols to purify pancreatic granules before separating membrane-bound and soluble granule fractions for bCHGB purification in detergents (Howell et al., 1969; Syversen et al., 1992). Luminal contents of the granules were released in three freeze–thaw cycles (Zhang et al., 2014). Western blotting showed that bCHGB existed in soluble and membrane-bound states at an ~50:50 ratio (Figure 3A; bands 1 and 2 correspond to mature and immature bCHGB, respectively) (Benedum et al., 1987; Pimplikar and Huttner, 1992). The two granule fractions were extracted in detergents and fractionated by ion exchange and size-exclusion chromatography before reconstitution into lipid vesicles. To assess the purity of bCHGB in vesicles, the proteins were re-extracted and fractionated by size-exclusion. The single gel filtration peak contained mature bCHGB of ~90 kDa as the only detectable band in Coomassie blue-stained SDS-PAGE, whose identity was confirmed by immunoblotting (WB, Figures 3B,C). In gel filtration, bCHGB in detergents was eluted at the same position as mCHGB, suggesting a dimer (Yadav et al., 2018).

A 70 kDa protein was detected by WB and determined as immature CHGB (Figure 3B). Because of this observation, we performed mass-spectrometry (MS) analysis at the UB CBLS proteomic/MS facility of the gel pieces between 50 and 80 kDa from the left lane of Figure 3B (near band II). Heat-shock protein (HSP70) was identified as the main contaminant. Band I on the same gel was identified as bCHGB dimer by WB. Quantification of the faint bands I and II and the main CHGB band in ImageJ revealed an estimated purity of ~99.3% for bCHGB (both band I and the main CHGB band; Figure 3D), making it feasible to assign measured activities in vesicles to bCHGB with similar high confidence as we did for mCHGB (Yadav et al., 2018; Jiang and Yadav, 2022). When the bCHGB vesicles containing proteins purified from either the soluble (blue line in Figure 3F) or membrane-bound (orange line in Figure 3F) granule fractions were floated in a density gradient, no protein was detected at the bottom loading position (Figure 3E), suggesting nearly 100% bCHGB reconstitution for either granule fractions. We thus concluded that once solubilized in detergents and purified, bCHGB from both the soluble and membrane-bound granule fractions appear biochemically equivalent and can be fully reconstituted into vesicles.

Next, we tested if the purified bCHGB retains anion channel functions by fusing the vesicles into lipid bilayers for electrophysiological recordings. A small amount of cholesterol was included because it supported the mCHGB channel activities (Yadav et al., 2018). The protein: lipid molar ratios (PLR) were kept at ~1: 10,000. The fused vesicles yielded small macroscopic currents from 5 to 15 channels per membrane patch (e.g., 5 channels for the recordings in Figure 3G). Without Ca^2+^ or Mg^2+^, the channels showed infrequent closing and opening events and no obvious inactivation, which is the same as mCHGB (Yadav et al., 2018). With stable channels and negligible leak currents, the reversal potential (−40.5 mV, Figure 3H) of the macroscopic currents in 150 (*cis*) / 30 (*trans*) mM KCl was used to estimate a permeation ratio of *P_Cl_ / P_K_* to be ~130. We also tried to patch blebbed vesicles by dehydration / rehydration-induced fusion, but the tiny vesicles failed to fuse well, likely due to the 5% cholesterol in these vesicles. The native bCHGB thus reconstitutes a highly selective anion channel. Given the consistent behaviors of 5+ channels and ~ 99.3% protein purity, the probability of any contaminating protein to produce such data was ~10^−6^ or less (Jiang and Yadav, 2022).

### bCHGB channels strongly favor Cl^−^ over Br^−^

**Figure 4:**
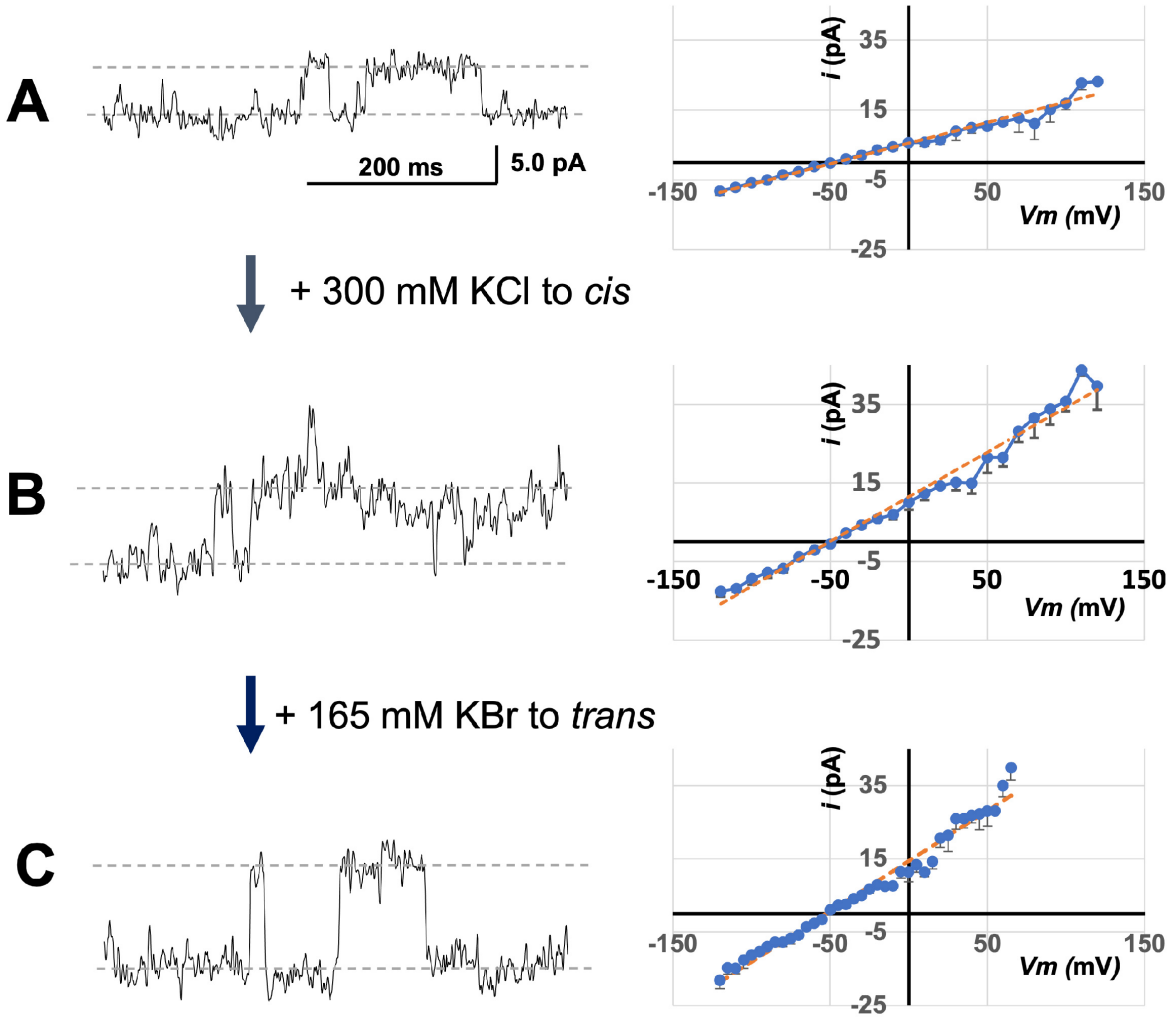
Sensitivity of bCHGB channels to varying anion compositions. The same channels in a lipid bilayer were first recorded in solutions containing (*cis/trans* mM: 150 KCl/30 KCl + 285 KBr) **(A)**. The left trace showed single channel events at 0.0 mV. Afterwards, 300 mM KCl was added to the *cis* side before the recording at 0 mV in panel **(B)**, and after that, 165 mM KBr was added to the *trans* side before the recording at 0 mV in panel **(C)**. Recordings on a series of Vm and from different membranes were obtained for analyses. The single channel currents <*i*> - Vm plots (right side) were obtained from 4 different sets of recordings. Errors: *s.d.*, *n* = 4. The average single channel currents at 0 mV were 5.4 ± 0.1, 10.2 ± 0.4, and 13.4 ± 0.2 pA in panels **(A–C)**, respectively. Linear fittings (orange dashed lines) were performed to estimate average single channel conductances *<g* > (~110 ± 7 pS, 196 ± 10 pS and 275 ± 14 pS in panels **(A–C)**, respectively; errors: *s.d.*, *n* = 4), and reversal potentials (*Erev* = −49 ± 1.7, −46.8 ± 1.2 and − 52.0 ± 0.2 mV in panels **(A–C)**, respectively; errors: *s.d., n* = 4). Their differences are all significant with *p <* 0.05. The single channel currents increased monotonically after KCl or KBr was added to the *cis* or *trans* side, and the *<g >* also changed accordingly.

Using lipid bilayers, we tested relative permeabilities of the bCHGB anion channels to Cl^−^ and Br^−^ by changing ionic compositions. After obtaining small macroscopic currents as in Figure 3G, we changed the solutions first to *cis/trans* (mM): 150 KCl/ (30KCl + 285 KBr) and measured single channel events (left of Figure 4A). This yielded an average single channel current (<*i*>) of ~5.4 pA at 0.0 mV. Then, we added 300 mM KCl to the *cis* side and observed a marked increase in the single channel current (left of Figure 4B), mounting to an average increase of <*i* > by 89% to 10.2 pA at 0.0 mV (left in Figure 4B; comparison to Figure 4A, *p* < 0.001). After that, 165 mM KBr added to the *trans* side caused a further increase of *i* at 0.0 mV, and its average to 13.4 pA (left of Figure 4C; comparison with Figure 4B, *p* < 0.0005). From linear fittings of the *<i > − Vm* plots (right sides of Figures 4A,C), the average single channel conductances, <*g*>, of the bCHGB channels in these three conditions were estimated to be ~110, 195, and 275 pS, respectively. When [Br^−^] in the *trans* side increased from 285 to 450 mM, the reversal potential decreased from −46.8 to −52.0 mV (*p* < 0.005), instead of being right-shifted to ~ −41 mV if Br^−^ permeation remained the same, suggesting Br^−^ and Cl^−^ interactions in the channel pore and related anomalous mole fraction effects (AMFEs) (Hille, 2001). Because of mutual interference, the permeability ratios of Cl^−^ to Br^−^ (P_Cl_: P_Br_) under different ionic conditions were difficult to determine using the Goldman–Hodgkin–Katz equation, but could be roughly estimated in a range of 7 to 20. The observed ionic composition-dependence in single channel currents, reversal potentials and P_Cl_: P_Br_ indicates two or more anion binding sites inside the pore, which will need structural evidence in the future, and demonstrates that the native CHGB channel strongly favors Cl^−^ over Br^−^, which agrees with the weak or no detectable Br^−^ permeation in the flux assays (Yadav et al., 2018).

With 450 mM KCl (*cis*) / (30 KCl + 450 KBr)(*trans*), Br^−^ introduced more frequent opening-closing events (Supplementary Figure 3A). We could recognize the subconductance states (marked as “sub”) at high positive and high negative potentials, and measure and compare macroscopic currents (*I*) and single channel currents (*i*) at different transmembrane potentials (*Vm*; Supplementary Figures 3B,C). Fitting of *I-Vm* and *i-Vm* plots generated reversal potentials of −52.6 and − 52.0 mV, respectively, showing a good agreement between the two and no significant effects from any leak currents. Compilation of available data defines an ion permeation sequence of the CHGB channel: F^−^ (1.2) ~ Cl^−^ (1.0) > > Br^−^ (~0.05 to 0.15) > K^+^ (~0.007 to 0.01), and a monotonic change of <*g >* with increasing [Cl^−^]: ~60 pS (15 mM), ~140 pS (160 mM) and ~ 275 pS (450 mM).

When millimolar Ca^2+^ or Mg^2+^ was present, mCHGB channels inactivated slowly at high positive and high negative potentials (Yadav et al., 2018). The same happened to the bCHGB channels with 2.0 mM MgCl_2_ when long voltage steps were used (5–10 s; Supplementary Figure 4A). Single exponential fitting of the data found an average inactivation time constant of ~2 s in the positive potentials of 70–100 mV, and ~ 1–4 s in the negative potentials of −60 to −100 mV (Supplementary Figure 4B). These estimates suggest that the inactivation is slow and happens in similar rates at both positive and negative potentials with weak or no voltage-dependence.

### Native CHGB channels show the same sensitivity to Cl^−^ and DIDS as the recombinant ones

**Figure 5:**
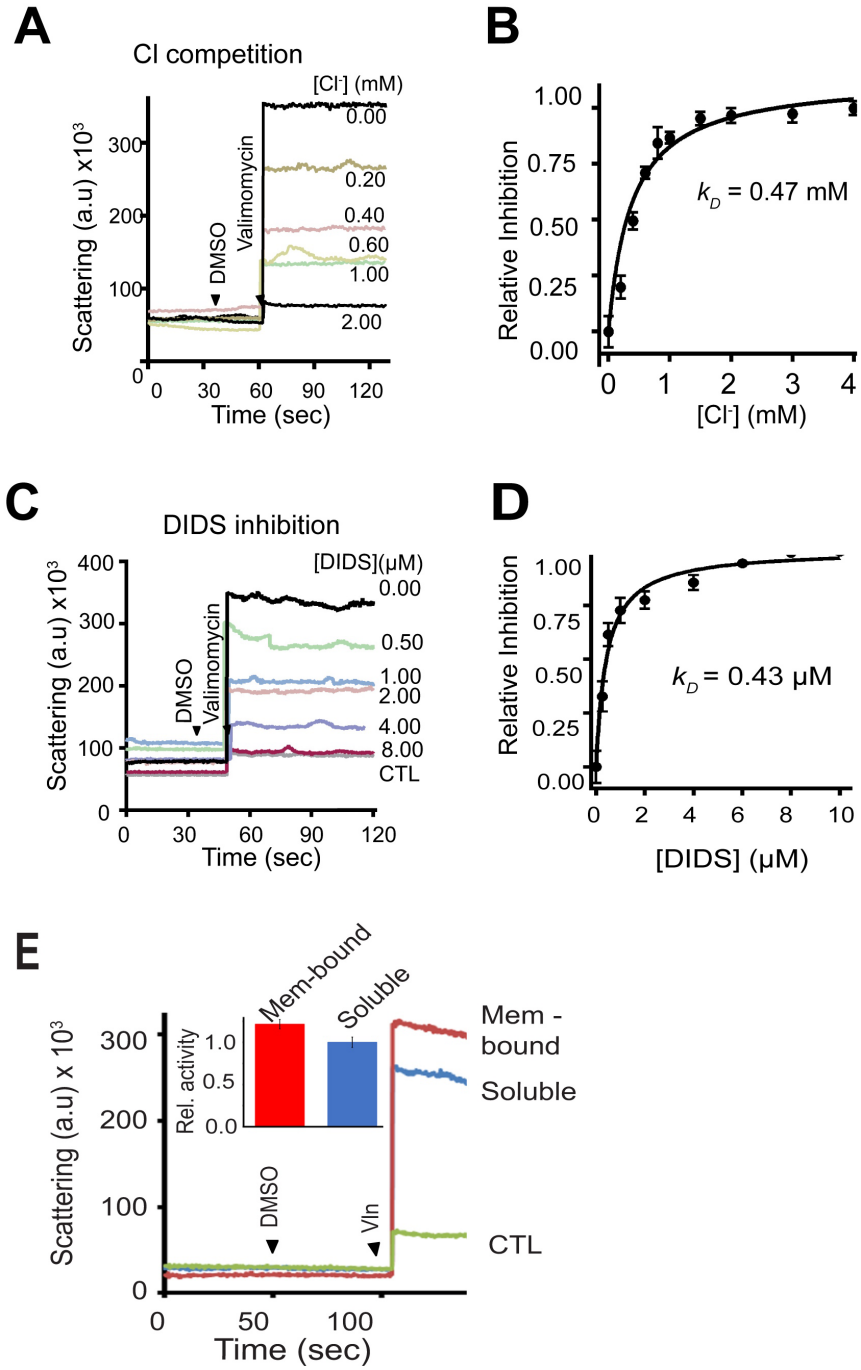
Billions of bCHGB channels tested by flux assays exhibit the same sensitivity to Cl^−^ and DIDS as the mCHGB channels. **(A)** Channel vesicles loaded with 300 mM KCl were analyzed with 300 mM KI plus varying amounts of KCl outside. Valinomycin was added to start the reaction, leading to changes in light-scattering. DMSO was added as control. **(B)** Inhibition of Cl^−^ efflux by extravesicular Cl^−^. Data from repeated experiments as in panel **(A)** (black circles; error bars: *s.d., n* = 3) fitted with a Hill equation, *I* = *1/*(1 + ([*L*]/*k_D_*)*^n^*), (black line) yielded a *k_D_* ~ 0.47 mM and a Hill coefficient *n* = 1.0. **(C,D)** Inhibition of Cl^−^ efflux by DIDS. Typical flux data as in panel **(C)** were plotted in panel **(D)** (black circles; error bars: *s.d. n* = 3) and fitted with a Hill-equation to yield a *k_D_* ~ 0.43 μM and a Hill coefficient *n* ~ 1.0. **(E**) Activities of bCHGB purified from soluble (blue) and membrane-associated (red) granule fractions were compared in the flux assays (a typical dataset from four repeats). Protein-less vesicles had no signal (CTL in green). Inset: Relative activity in the flux assays of two forms of native bCHGB protein.

The recombinant mCHGB channels are sensitive to Cl^−^ (due to change in chemical potentials) and DIDS (due to physical binding) (Nobile et al., 2000). To better assess the channel sensitivity to Cl^−^ and DIDS, we performed light scattering-based flux assays, which sampled billions of bCHGB channels each time (Yadav et al., 2018; Yadav and Jiang, 2020). We titrated Cl^−^ and DIDS on the extravesicular side and found that increasing [Cl^−^] from 0.0 to 2.0 mM (Figure 5A) gradually decreased the flux signals, revealing a driving force change with an apparent *k_D_* = 0.47 mM (Figure 5B), nearly the same as the recombinant mCHGB channel (*k_D_* ~ 0.49 mM) (Yadav et al., 2018). Similarly, DIDS, an anion channel blocker, inhibited bCHGB channel activity with an apparent *k_D_* ~ 0.43 μM and a Hill coefficient, *n* ~ 1.0 (Figures 5C,D), also almost the same as the mCHGB channel. As our channel is pure and the measured DIDS sensitivity is high and close to the NPPB-affinity (~50 nM) for the CLC channels, we did not test other Cl^−^ channel-blocking compounds here. The native bCHGB channel is thus pharmacologically equivalent to the recombinant mCHGB channel, suggesting that an intrinsic property of pure CHGB proteins, whether native or recombinant, is to reconstitute a highly-selective anion channel with a *P_Cl_: P_Br_* ~ (7–20): 1, which differs from *P_Cl_: P_Br_* < = 1 of most known Cl^−^ channels, but agrees with differences in ion size (1.7 vs. 1.9 Å) and thermodynamic energy for dehydration (enthalpy and entropy changes) as discussed before (Hille, 2001; Yadav et al., 2018).

The dimorphic nature of bCHGB should allow its soluble form to reconstitute Cl^−^ channels in the membrane equally well after detergent extraction and membrane insertion. Indeed, the flux assays of bCHGB vesicles made from the “soluble” and “membrane-bound” granule fractions both yielded strong Cl^−^ flux signals (Figure 5E). By contrast, protein-free vesicles yielded no signal (CTL); so was recombinant mCHGBΔMIF, a nonconducting mutant. The soluble form of bCHGB thus retains the capacity of reconstituting Cl^−^ channels, after it undergoes potential conformational changes when being shifted into detergent micelles and then lipid bilayers. In bilayers, its channels showed similar properties as those made of bCHGB from the membrane-bound fractions.

### CHGB *in situ* is dimorphic due to its strong interactions with biomembranes

**Figure 6:**
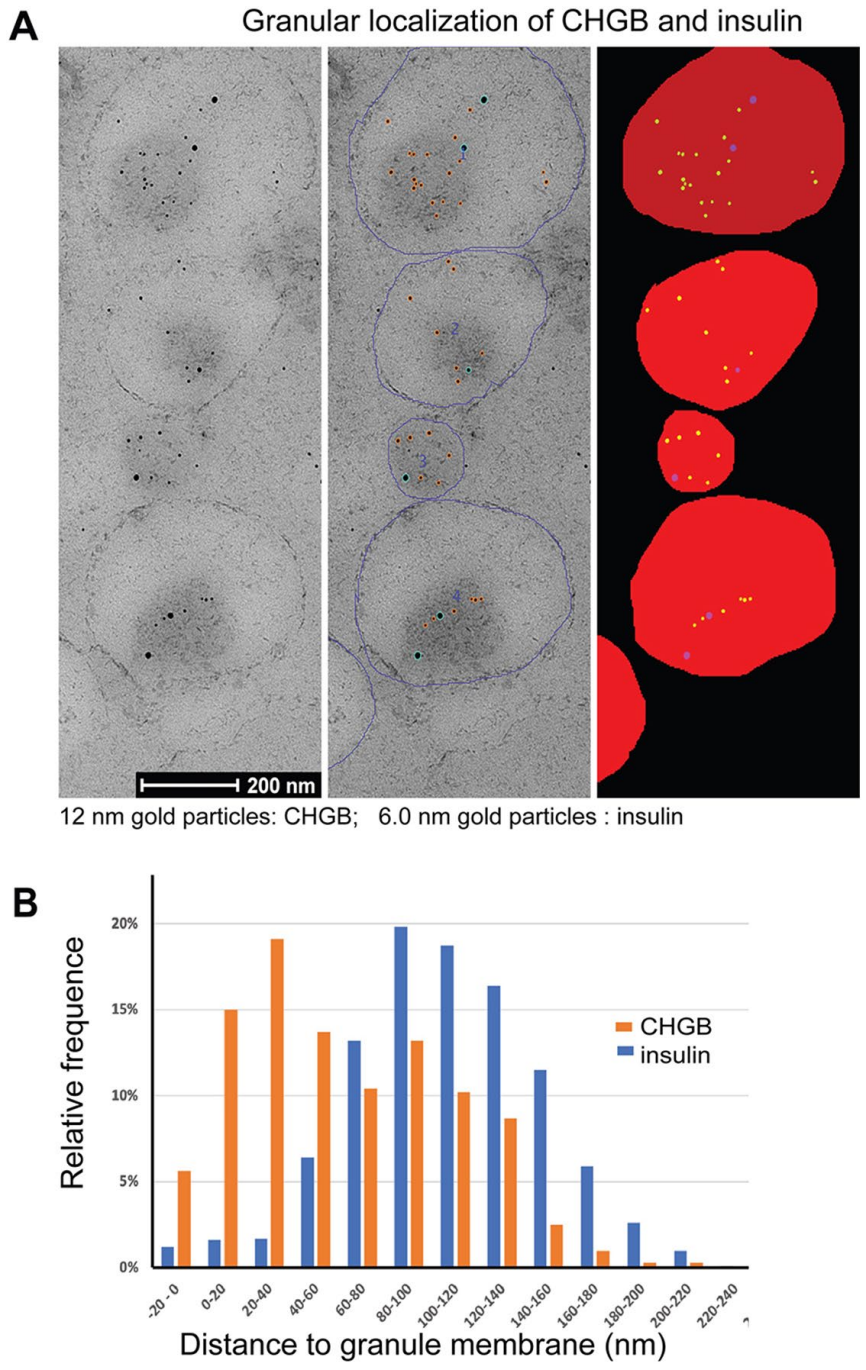
CHGB shows two distinct forms in secretory granules of primary β-cells from rat pancreas. **(A)** Typical high pressure freezing (HPF) / immuno-EM images on the left panel show distribution of CHGB and insulin inside DCSGs of a β-cell. CHGB was marked with 12 nm gold particles while insulin by 6 nm ones. Antibodies were tested in different concentrations to find one that minimizes nonspecific signals in other cellular compartments. Individual granules were demarcated (blue circles in the middle) and then filled for automatic detection of gold particles and quantitative analysis of their distances to the closest granule membranes (right panel). **(B)** Histograms of CHGB- and insulin-labeling nanogold particles in their distances to the closest granular membranes. Data from three separate experiments showed the same distributions and thus were pooled together for statistical analysis. A major fraction (~40%) of CHGB-labeling gold particles are within 40 nm of the granular membrane as compared to the insulin-labeling ones that are mostly (>95%) 80–140 nm away from the nearest membranes.

Our biochemical data and fluorescence imaging data from cultured cells are all consistent with the idea that the native CHGB is dimorphic. How about CHGB in tissue cells? Because regular fluorescence microscopy is limited in spatial resolution (~200 nm), we performed high-pressure freezing / immuno-electron microscopy (HPF /immuno-EM) to analyze *in situ* distribution of rat (r) CHGB in β-cell granules. Insulin was chosen as a negative control because it is soluble and concentrated to the core of DCSGs due to crystals of its Zn^2+^-coordinating hexamers. HPF/immuno-EM can resolve individual bilayer membranes (~4 nm) well and is superior in preserving cellular structures and revealing genuine, physiological distribution of target molecules. Dual immuno-gold labeling of CHGB and insulin was performed on high-pressure-frozen and Lowicryl HM20 freeze-substituted islets of Langerhans from rat pancreas (van Weering et al., 2010; Fava et al., 2012; van Weering et al., 2012). CHGB-antibodies were conjugated with 12-nm gold particles and insulin antibodies with 6.0-nm ones (left in Figure 6A). To minimize nonspecific labeling, interferences between two labeling antibodies or potential labeling-induced structural changes, different concentrations of antibodies were first tested by serial dilution. The lowest concentrations that gave decent labeling in granules without detectable labeling in other intracellular compartments were then chosen (e.g., Supplementary Figure S5A).

For quantitative analysis, the circumference of each granule was marked out (middle in Figure 6A) before an automated plugin for FiJi was used to detect 6 and 12 nm gold particles and fill the granular volume with an extension to the outer edge by 20 nm (Figure 6A, right column). This procedure allowed automatic determination of the distance of a gold particle to the nearest membrane of its residing granule. A total of 324 granules from 3 independent experiments were analyzed. Because no differences in distance distribution were observed between separate experiments, all data from three experiments were pooled for analysis to gain strong statistical power by the large number theorem. The measured average granule diameter is ~196 nm (Supplementary Figure 5B), close to past observations (Fava et al., 2012). When the measured distances were cast into 20-nm bins (Figure 6B), histograms showed clearly a two-modal distribution of rCHGB, but a mono-modal distribution of insulin. The two apparent Gaussian peaks of the rCHGB distribution are centered at ~25 nm and ~ 110 nm, respectively. The first peak shows significant signals outside granule edges (6% in the −20 to 0 nm bin). In contrast, insulin labeling in the −20 to 0 nm bin is negligibly small (<1.5%). The second peak of rCHGB overlaps closely with the only Gaussian peak of insulin distribution at ~117 nm. The difference between the first and second rCHGB peaks is statistically significant (*p* < 0.00001). Contrary to insulin being inside the granules, ~40% of rCHGB is within 40 nm (inside or outside the granule edges) of granule membranes, consistent with the equal distribution from the biochemical data in Figure 3A. Given the sizes of the nanogold-labeled secondary antibody (14.5 nm), the linker (~1 nm), the primary antibody (14.5 nm) and the part of CHGB outside the membrane (~4 nm), the major peak of the nanogold-labeled membrane-bound CHGB is expected to be less than ~40 nm (=5 + 14.5 * 2 + 12 * 0.5) from the membrane on the luminal side. Alternative orientations of the antibodies projected toward the outside are expected to keep the gold particles less than 26 nm (= 14.5*2–5 + 12*0.5–4) away from the granule outer edges (the −20 to 0 nm bin; 4 nm for bilayer thickness). These estimates match with the experimental data. Even though it was impractical to resolve individual CHGB proteins in the HPF samples, let alone transmembrane segments, the CHGB-labels detected in the outer rim of the granule membranes reflect more probably membrane-inserted rCHGB, considering its known resistance to dissociation under very harsh conditions, except detergents (Pimplikar and Huttner, 1992).

### A native bCHGB dimer encloses a central pore with end openings

The above results showing the membrane-residing nature of the native CHGB *in situ,* which reflects the current state of the art in studying membrane-integral proteins in native membranes, are limited in spatial resolutions of 3–4 nm, and therefore cannot discern a partially membrane-inserted or one leaflet-deep re-entry protein from a truly membrane-spanning one. Our analysis of the channel functions on the cell-surface (Figure 1A) and in lipid vesicles or planar bilayers (Figures 1, 3, 4) demonstrated necessarily a membrane-spanning state of the native CHGB in order not to violate the laws of physics behind ion conduction. To reveal the structural basis for the CHGB channel, we used single particle cryo-EM to reveal the physical nature of the ion-conducting pore enclosed by a CHGB dimer in detergents and assessed if the protein is structurally suitable to form a channel. Because each tetrameric channel contains two dimers with two pores, the dimer in detergents expectedly contains one pore (Yadav et al., 2018).

**Figure 7:**
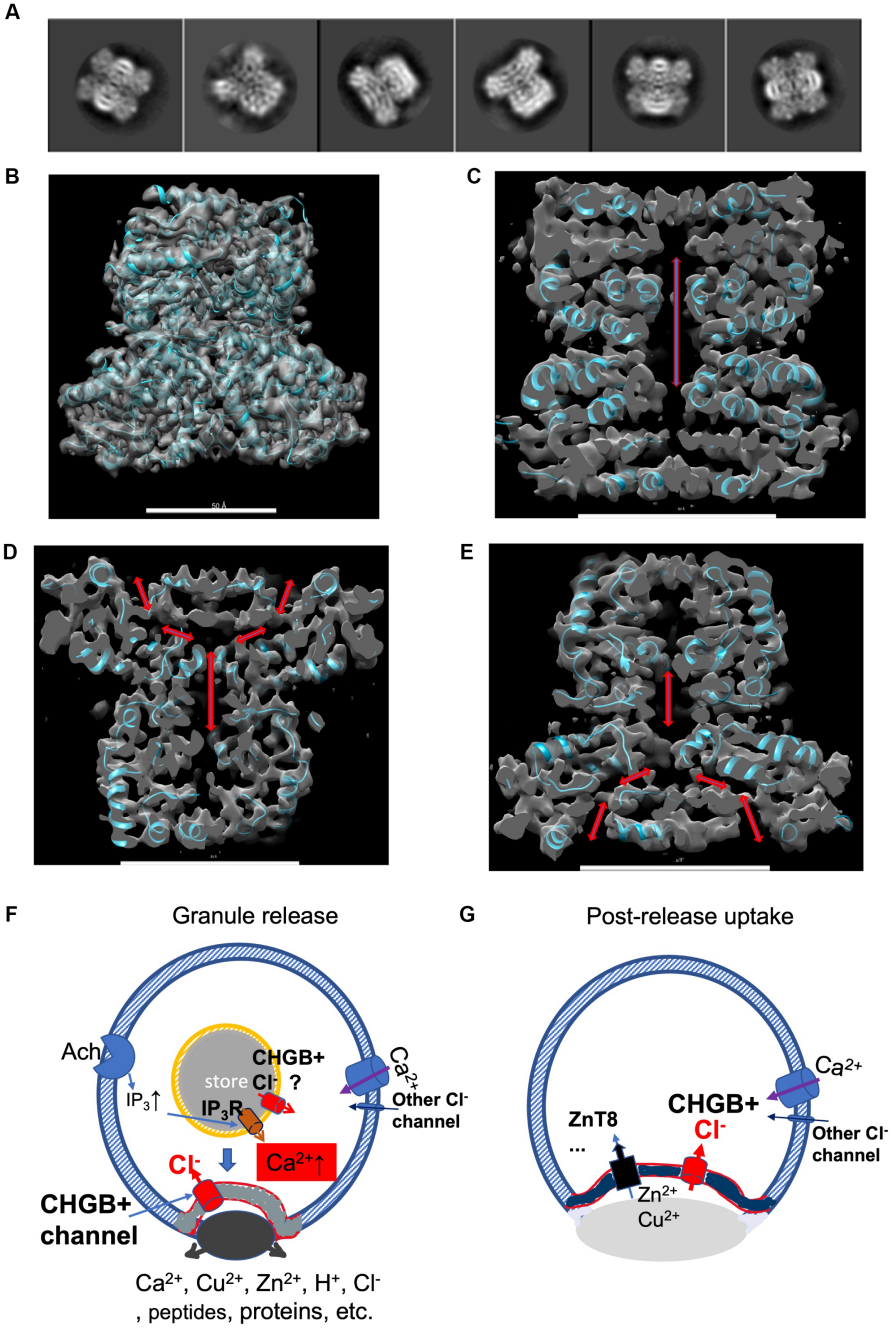
A cryo-EM structure of bCHGB dimer reveals a central pore with end openings, suggesting potential physiological roles of the CHGB+ channels. **(A)** Good 2D class averages from cryo-EM images of bCHGB dimers. **(B)** A map of a nominal 3.5 Å resolution (FSC = 0.143) viewed toward its C2 axis in the vertical direction with a poly-Ala model (cyan ribbons). **(C)** A central section showing a pore (double arrow heads). (D&E). Central sections after the map was rotated counterclockwise **(D)** or clockwise **(E)** by 45 degrees to show two side openings of the central pore to the top (arrowheads in **D**) or the bottom (arrowheads **E**), respectively. Scale bars in panels **(B–E)**: 5.0 nm. **(F)** During granule release, the CHGB+ channels show up in the plasma membrane in small puncta (grey) as a dominant Cl^−^ conductance. If active in the DCSGs, they might facilitate IP_3_-triggered Ca^2+^ release. As an example, Ach receptors coupled to G-proteins lead to downstream production of IP_3_. On the cell surface, the CHGB+ channels may provide a positive feedback for Ca^2+^-triggered exocytosis. **(G)** After granule release and diffusion of the dense core granules, CHGB+ channels may support the uptake of divalent cations (such as Zn^2+^ or Cu^2+^) by transporters or channels, such as ZnT8, ZIP-6,-7, −13, or − 14, etc. Maps are available at wwPDB with a code of EMD-36579.

Previously, a negative-stain map of the mCHGB dimer was refined to ~10 Å with a cryo-EM dataset after multi-round classifications (Yadav et al., 2018), suggesting a well-packed structure, instead of a disordered monomer as predicted by AlphaFold2.^1^ Here, we used native bCHGB for cryo-EM studies because of its high purity and stable dimers (Figure 3). We collected a small dataset in a Glacios/Falcon 4 system at HWI that was benchmarked at 2.0 Å, and found a few classes showing strong C2 symmetry among many ones showing particles of similar shapes (Supplementary Figure 6; Figure 7A). *Ab initio* reference maps from selected particles in cisTEM and cryoSPARC took the same shape as the ~10 Å map of the mCHGB dimer, (Punjani et al., 2017; Yadav et al., 2018; Lucas et al., 2021) strengthening the confidence in deep classification and refinement. A homogeneous dataset of 35,000 particles was sorted out to calculate a 3D map at a nominal resolution of ~6.8 Å (FSC = 0.143) in Relion 3.1 (Scheres, 2015). Independent analyses in cisTEM and cryoSPARC produced the same results. At a higher threshold, the map showed rod-like features, indicating alpha-helices (Supplementary Figures 7A,B), a central opening, and a putative membrane-inserted state (Supplementary Figure 7C).

To recognize the pore better, we improved the cryo-EM map to a nominal 3.5 Å resolution by using a larger set of ~88,500 multi-frame movies collected in a 300 kV Titan Krios, mainly at the Purdue University. After processing, we achieved a resolution that allows good recognition of main chain residues and secondary structures (Figure 7B; Supplementary Figure 8). Local resolutions in the core of the map (~3.3 Å) are better than that in the periphery (~5.0 Å), suggesting room for improvement (Supplementary Figures 8D,E). Without any prior structural information, the main chain residues detected by both DeepTracer and Model_Angelo agreed relatively well with each other (Pfab et al., 2021; Jamali et al., 2023), better than other conventional tools for model building, probably due to the current resolution of the experimental map. A partial model without details of all side chains (cyan ribbons in Figure 7B) matched with the densities sufficiently well, and was used to help recognize the enclosed cavity and the end openings (arrows in Figures 7C–E). Along its C2 axis, a CHGB dimer encloses a central pore that opens to top and bottom ends, and is more than 65 Å in length and more than 6 Å in diameter.

## Discussion

### Native CHGB forms anion channels in granule membranes and is delivered to cell surface upon regulated secretion

The foregoing experiments demonstrate that native CHGB is dimorphic in secretory granules and able to reconstitute anion channels with a large single channel conductance and high anion selectivity as well as pharmacology similar to the channels made of the purified recombinant mCHGB. More importantly, the native CHGB dimer contains an internal cavity (pore) suitable to act as a channel, supporting two pores in a tetrameric CHGB channel. Therefore, it is plausible that the CHGB-dependent anion conductances on the plasma membrane that emerged in response to membrane depolarization represent the delivery of the CHGB+ channels via regulated secretion.

Our findings reveal two unique phenomena of regulated secretion – (1) significant cell-surface anion conductances in parallel to granule release, which differs from synaptic vesicle release and immune degranulation, and (2) CHGB, a dimorphic protein that exists in both soluble and membrane-bound states, directly contributes to such conductances, either on its own or in combination with other proteins. Our data support that the CHGB+ conductances (~28 nS) are much larger than other channels, including possible Ca^2+^-activated Cl^−^ channels. Our cryo-EM structure of the bCHGB dimer demonstrates its pore-forming capacity, making it much more plausible that CHGB directly forms the anion channel pore that mediates the conductances seen on the cell surface, even in the cases that other subunits are involved. This is further supported by the finding that native CHGB proteins of ~100% purity can reconstitute into anion channels just like the recombinant mCHGB. Therefore, PTMs do not alter the channel-forming capacity of native CHGB. Neither are other proteins required for CHGB to form the channel, although it is likely that association with other protein partners may alter (or modulate) the properties of the CHGB+ channels. Additional evidence supporting the roles of CHGB in the anion conductances delivered by regulated secretion comes from the observations that both CHGB and vATPase puncta appeared on the cell surface in response to depolarization. Regarding the possibility that CHGB diffused out of the secretory granule regions on the cell surface might facilitate the opening of CHGB-free anion channels pre-existing there (like CACCs) and/or being delivered there during regulated secretion via non-granular vesicles, diffusion of CHGB tetramers out of the puncta in plasma membranes (longer than a few minutes instead of <1 s) or its soluble dimers or oligomers out of the dense, viscous phase of a dissolving granule through a fusion pore (>3 s) is too slow to support the quick emergence of anion channels (Figure 1A; Pimplikar and Huttner, 1992; Weiss et al., 2014). The existence of this group was further negated by the dominant negative effects of a CHGB deletion mutant (Yadav, companion paper). Certainly, should these hypothesized channels exist, they would be called CHGB+ channels, too.

Without full atomic details, a partial structural model based on the 3.5 Å cryo-EM structure reveals that the dimorphic CHGB takes a noncanonical topology, differing from poly-topic alpha helical bundles or beta-barrels of a conventional monomorphic ion channel (Jiang, 2021). It remains to be determined which residues line the pore, which ones dictate ion selectivity and fast permeation, how two dimers come together to form a stable tetrameric channel, and whether a dimer undergoes significant conformational changes during tetramer formation. These will require a better model with side chains of key residues resolved so that mutations can be introduced for direct tests. Our prior data suggested that a subconductance state of ~50% of the full opening likely comes from one open pore (Supplementary Figure S3A; Yadav et al., 2018) and a CHGB+ channel may contain two pores that can be gated both jointly and separately, as in the double-barrel architecture of a CLC0 channel (Middleton et al., 1996).

### Potential physiological roles of CHGB+ channels near or at the cell surface

Anion channels delivered by regulated secretion may have at least two possible roles. First, these channels can facilitate granule release. At rest, a basal level of granule release may put a very small number of CHGB+ channels on the cell surface (Figure 2C). These channels may help compensate for local charge imbalance in the early phase of Ca^2+^ influx (Obermüller et al., 2010) (Figure 7F). When more granules are released, more CHGB+ channels coming to the cell surface can accelerate Ca^2+^ influx. Assuming a local volume of ~40 femtoliters at an exocytosis site (~1% of total cell volume), an increase of [Ca^2+^] to 10 μM will bring in 2.5 × 10^5^ calcium ions near the release site, creating a charging effect (estimated up to 0.6 volts under an assumption that the charges are localized around a granular puncta in a circular area of 4 μm in diameter on the cell surface). Such a strong effect needs neutralization by local ion flux, such as Cl^−^ influx. Inside DCSGs, if the CHGB+ channels become active right before granule exocytosis, they can also facilitate Ca^2+^ release from secretory granules (such as IP_3_-triggered release in Figure 7F). These two processes together can promote local Ca^2+^ spikes, as well as a fast and even concerted release of RRGs, which probably happens during the 1st phase of fast insulin release (within 5–10 min) after a glucose challenge (Barg et al., 2002). Calcium waves via gap junction channels between β-cells may synchronize insulin release in this phase, too (Carvalho et al., 2012). To probe the anion channels in DCSGs or granules in general, direct electrophysiological recordings or other assays coupled to channel activity (Yadav et al, companion paper) and Ca^2+^ measurements right at the granule release sites will be needed in the future.

Second, the CHGB+ channels on the cell surface may help restore sudden loss of divalent cations (Figure 7G). DCSGs contain high concentrations of divalent cations (Ca^2+^, Zn^2+^, and/or Cu^2+^). A quick release of ~50 RRGs in a short time only causes a loss of cell volume by ~0.5 femtoliters, which is negligibly small (~0.01%) in comparison to the total volume of a typical β-cell (~5 picoliters); however, a cell may lose a significant fraction (~ 1–5%) of its intracellular Ca^2+^, Zn^2+^ or Cu^2+^. For example, at an insulin release site the extracellular Zn^2+^ concentration may suddenly rise to a μM level when the DCSGs are dissolved (Li et al., 2011). A quick reuptake of Zn^2+^ is thus energetically favored for the cell. If Zn^2+^ diffuses into the cytosol via a transporter, such as ZIP-6,-7, -13, or -14 on the cell surface or ZnT8 in the granule membranes (Chimienti et al., 2006; Xue et al., 2020), the cell-surface CHGB+ channels are rightly positioned to neutralize positive charges accumulated inside due to Zn^2+^ reuptake.

These two roles of CHGB+ channels may be compensated for by other channels or transporters in the *Chgb−/−* mice, or may be tolerated by cells spending more energy in ion homeostasis and regeneration of secretory granules, which could in part explain weak phenotypes of the knockout mice (Díaz-Vera et al., 2010; Obermüller et al., 2010; Zhang et al., 2014). Nonetheless, the defects may bring in more severe consequences if a knockout mice faces a shortage of energy. Further investigations are needed to assess such possibilities.

### CHGB+ channels are more selective than channels made of other dimorphic proteins

While CHGB+ channels play physiological roles, other dimorphic proteins form non-selective channels to exert pathogenic or cytotoxic effects. For example, C-type lectins released from small intestine epithelial cells reconstitute hexameric pores in membranes of *G^+^* bacteria to kill the germs (Mukherjee et al., 2009, 2014). VopQ, a pathogenic effector protein of the *Vibrio* species, is released inside infected cells to puncture lysosomal or vacuolar membranes by forming non-selective channels, which then causes cell demise (Sreelatha et al., 2013, 2015). Other examples include non-selective CLIC proteins (Tulk et al., 2000; Ashley, 2003; Edwards, 2010; Gururaja Rao et al., 2017; Hossain et al., 2019), membrane-attacking C5b/C6-9 complexes (Michaels et al., 1976; Young and Young, 1990; Zalman and Muller-Eberhard, 1990; Bayly-Jones et al., 2017; Menny et al., 2018; Parsons et al., 2019), colicins (Schein et al., 1978; Weaver et al., 1981; Kienker et al., 2000), Gasdermin D N-terminal domains (GSDMD) in pyroptosis (Chen et al., 2016; Ding et al., 2016; Liu et al., 2016; Ruhl and Broz, 2016; Ruan et al., 2018), BAK or BAX in mitochondria (Antignani and Youle, 2006; Cosentino and Garcia-Saez, 2017; Uren et al., 2017a,b), phosphorylated MLKL channels for necroptosis (Su et al., 2014; Wang et al., 2014; Zhang et al., 2016; Zhang and Han, 2016; Shrestha et al., 2019), microbiocidal defensins (Hristova et al., 1996; Zhang et al., 2010; Awang and Pongprayoon, 2018), etc. The nonselective pores may break down transmembrane ionic gradients and result in loss of nutrients, ATP and other metabolites, or even small proteins. Contrastingly, the CHGB+ channels need high selectivity to prevent unintended loss of intracellular organic anions and block rushing-in of extracellular anions after being delivered to the cell surface. The stringent selectivity thus prevents breakdown of cation or organic anion gradients across granular or cell membranes, and minimizes post-exocytotic loss of cytosolic anionic metabolites.

Among all dimorphic proteins that form ion channels, CHGB is so far the only one showing a strong ion selectivity (Yadav et al., 2018), which agrees with the lack of detectable permeability to all cations (except protons) of the isolated chromaffin granules (Johnson and Scarpa, 1976). Compared to other Cl^−^ channels, such as CLC family or ANO-1/2 channels, CHGB bears the highest selectivity for Cl^−^ over Br^−^ (Fahlke, 2001; Jentsch and Pusch, 2018). The Br^−^ and Cl^−^ interactions implicated by our experiments (Figure 4) suggest that the channel likely contains two or more anion binding sites, with Br^−^ binding interfering with Cl^−^ permeation, and vice versa. How the interplay between the ions affects the permeation of CHGB+ channels warrants further investigations.

### CHGB+ channels differ from other ion channels assigned to secretory granules

Previously, a large-conductance Ca^2+^-activated K^+^-channel (BK), a few other K^+^ channels and a 250 pS Cl^−^ channel (450/150 mM Cl^−^) were assigned to isolated chromaffin granules (Hordejuk et al., 2006). However, no significant permeability of cations other than proton was observed in isolated chromaffin granules four decades ago (Johnson and Scarpa, 1976; Johnson et al., 1982). These two reports could be reconciled by considering possibly low sensitivity of the flux assays (Johnson and Scarpa, 1976), or presence of biochemical impurities in the isolated granules (Hordejuk et al., 2006). Our whole-cell recordings (Figure 1) detected nearly exclusively an increase in anion currents, suggesting that in secretory granule membranes the anion channels are much more abundant than any of the proposed K^+^ channels or any unknown nonselective channels. The large conductance bCHGB channels (~275 pS/450 mM) may be related to the 250 pS Cl^−^ channel, given the sensitivity of its conductance to varying anion composition (Figure 4). Additional studies with highly specific inhibitors or genetic manipulation may be performed to examine the 250 pS channel further or define its genetic identity. Further, Kir6.1, ATP-sensitive K^+^/Cl^−^ channels, and CLC-1/2 channels were reported from zymogen granules (Thevenod et al., 2000; Kelly et al., 2005). The recorded Cl^−^ channels in these granules behaved differently from the same channels recorded in cultured cells (Thevenod et al., 2000; Kelly et al., 2005; Guzman et al., 2013; Stolting et al., 2014). The CLC channels have much smaller single channel conductances than the CHGB+ channels and may serve different roles when coexisting with the latter in cell membranes.

## Conclusion

The anion channels delivered to the cell surface via secretory granule release have CHGB as an essential component (CHGB+), endowing a new biochemical marker and a strong electrical signature to the regulated secretory pathways in high-order organisms. In DCSGs, native CHGB is dimorphic, existing in both soluble and membrane-bound forms, and both forms can reconstitute anion channels in membranes. The CHGB dimer encloses a long central pore with openings to opposite ends, probably allowing multiple ion binding sites to account for its unprecedented anion selectivity. The CHGB+ channels may function in supporting Ca^2+^-dependent, concerted granule release or post-release reuptake of divalent cations. Potential functions of CHGB+ channels in cellular processes of regulated secretion still await future investigations. The highly conserved CHGB orthologs in vertebrates probably play similar functions.

## Data availability statement

The original contributions presented in the study are included in the article/Supplementary materials, further inquiries can be directed to the corresponding authors.

## Author contributions

Q-XJ designed and oversaw the studies, performed bilayer recordings, analyzed results with all co-authors, and analyzed all data with others in the group and wrote the manuscript with contributions and/or comments from the group. GY and Q-XJ designed and performed all molecular cloning, biochemical studies, and cell-based experiments, and analyzed acquired data. HW, QW, and MZ conducted electrophysiological experiments in PC-12 cells and analyzed data together with Q-XJ. JO, SC, and PV did HPF-immuno-EM and data analysis with assistance from GY and Q-XJ. FQ helped with the biophysical analysis of bilayer recordings as well as trials of recordings from fused vesicles. All authors contributed to the article and approved the submitted version.

## Funding

The work in the Jiang lab was supported by NIH (R21GM131231, R01GM111367, and R01GM093271 to Q-XJ), *CF* Foundation (JIANG15G0 to Q-XJ), Welch Foundation (I-1684 to Q-XJ) and CPRIT (RP120474 to Q-XJ), and by an AHA National Innovative Award (12IRG9400019 to Q-XJ), an NIGMS EUREKA Award (R01GM088745 to Q-XJ), a pilot grant from the Office of Research at the University of Florida (to Q-XJ), and startup funds from the University of Texas Southwestern Medical Center, the University of Florida and the Hauptman-Woodward Medical Research Institute. Some of the experiments reported here were performed in a laboratory constructed with support from NIH (C06RR30414). Besides EM facilities at home institutions, the cryo-EM studies in the Jiang lab were supported by SEM4 consortium at the Florida State University (grant #1U24GM116788) with Q-XJ as one of the MPIs, the National Cancer Institute’s National Cryo-EM Facility (NCEF) at the Frederick National Laboratory for Cancer Research under a contract HSSN261200800001E, the NIH-funded consortium at the Purdue University (U24GM116789) with Q-XJ as one of the co-PIs as well as facilities at the Case Western Reserve University and the New York Structural Biology Center under the support of the NIH Common Fund Transformative High Resolution Cryo-Electron Microscopy program (U24GM129539) and by grants from the Simons Foundation (SF349247) and NY State Assembly Majority.

## Conflict of interest

The authors declare that the research was conducted in the absence of any commercial or financial relationships that could be construed as a potential conflict of interest.

## Publisher’s note

All claims expressed in this article are solely those of the authors and do not necessarily represent those of their affiliated organizations, or those of the publisher, the editors and the reviewers. Any product that may be evaluated in this article, or claim that may be made by its manufacturer, is not guaranteed or endorsed by the publisher.

## Footnotes

^1^ https://alphafold.ebi.ac.uk/

## Abbreviations

CHGB: chromogranin B
ISG: immature secretory granule
DCSG: dense-core secretory granule
TGN: *trans*-Golgi network
CLC: chloride channel / transporter
RRG: readily releasable granule
MIF: membrane-interacting fragment
CHGB-MIF: a membrane-interacting fragment at the C-terminal half of CHGB
CHGBΔMIF: CHGB deletion mutant lacking its MIF
cryo-EM: cryo-electron microscopy or cryogenic EM.
