# Supplementary material for: Chromogranin B (CHGB) is dimorphic and responsible for dominant anion channels delivered to cell surface via regulated secretion
Source: Front Mol Neurosci. 2023 Jun 26;16:1205516. doi: 10.3389/fnmol.2023.1205516 (PMC10330821; doi:10.3389/fnmol.2023.1205516)
Supplement: Supplementary file 1 [file Presentation_1.pdf]

Supplementary Information

List of items:

I. Supplementary Figures

II. List of reagents

III. Supplementary References

I. Supplemental Figures

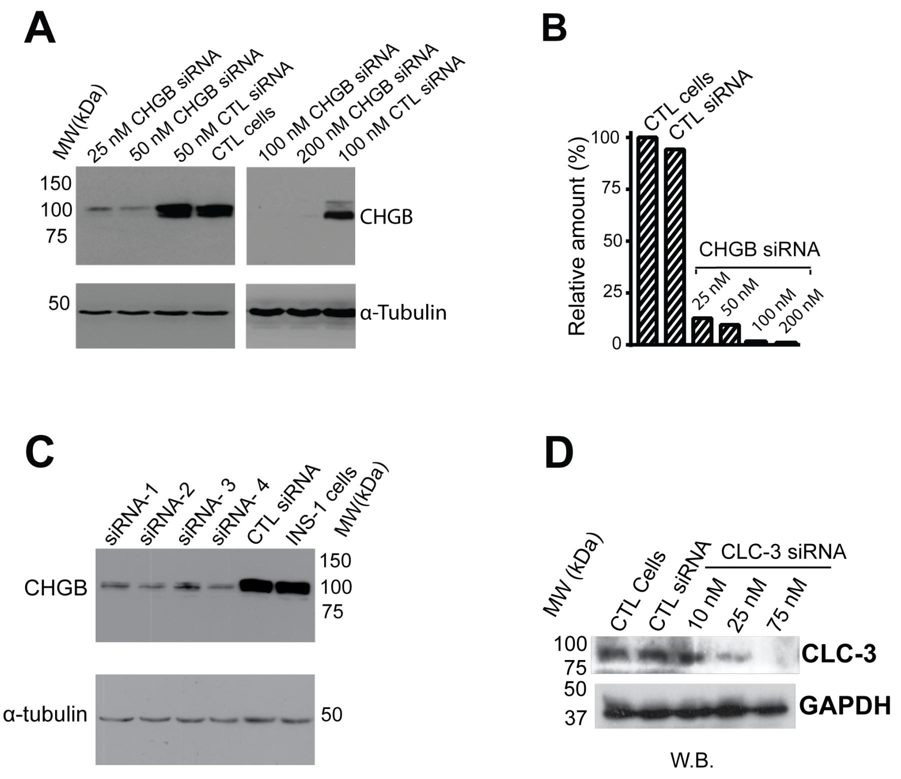

supp. Fig 1

Supplementary Figure 1. Test of siRNAs for CHGB and CLC-3.
(**A**). Dose-dependent knockdown of CHGB expression in INS-1 cell. Western blot of CHGB from cells transfected with different concentrations of CHGB-targeting siRNAs or control sequence-scrambled siRNAs (scRNAs), or control cells (CTL). Approximately twenty micrograms of protein from cell lysates were used for immunodetection. 100 nM siRNAs were selected for other experiments. (**B**). Relative amounts of CHGB proteins from the same number of cells (based on the total protein and the loading control) under different conditions. Three repeated experiments showed the same trend of does-dependence. (**C**). Four different siRNA molecules in the CHGB-specific siRNA mixture were compared

individually at 50 nM. Specific effects were seen for all four with siRNAs 2 and 4 being more effective than siRNAs 1 and 3. The siRNAs 2 and 4 were used separately or in combination to minimize off-target effects. (**D**). Dose-dependent knockdown of CLC-3 by siRNAs. 75 nM is suitable for experiments.

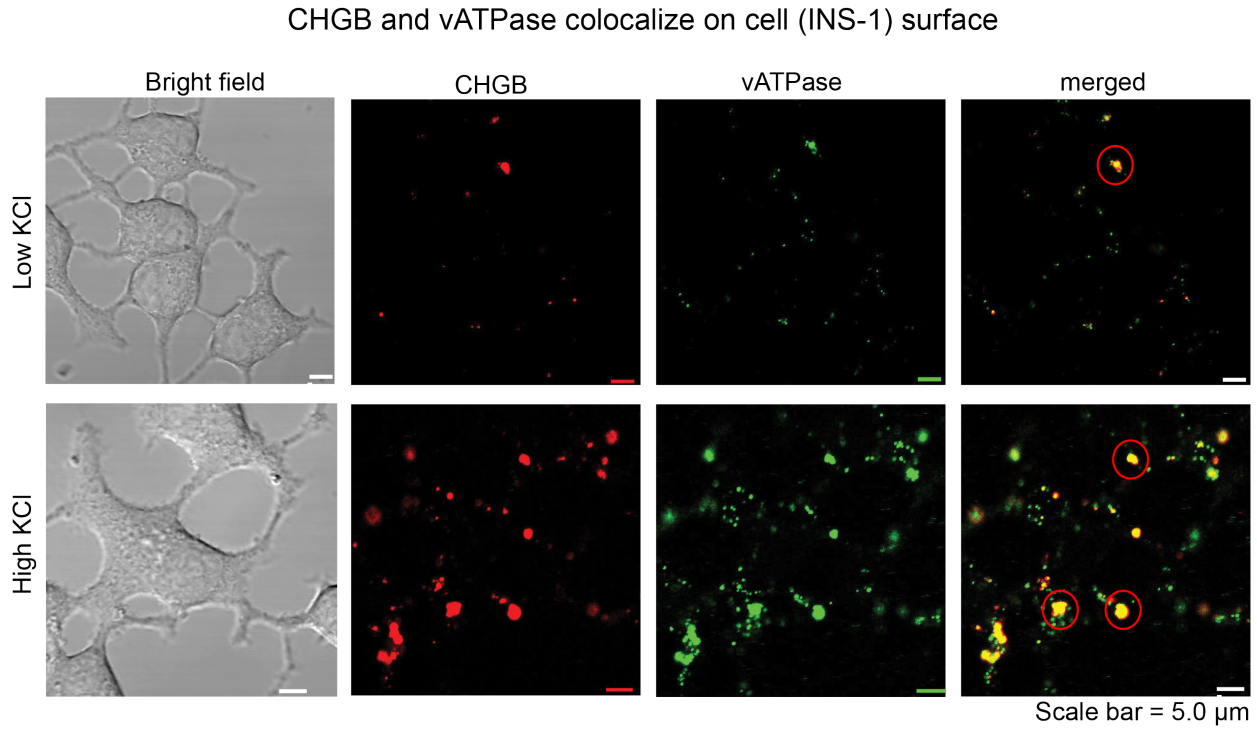

Supplementary Figure 2 Colocalization of CHGB and vATPase on cell surface after granule release. INS-1 cells were treated with 4.8 mM (Top row) or 55 mM KCl (Bottom row) at 37ºC for 15 minutes before being transferred to ice. The cells were blocked with 2 % BSA for 15 minutes on ice and probed with the anti-CHGB antibody and an Alexa-647-conjugated secondary antibody and with the anti-vATPase A2 antibody and the Alexa-488 conjugated 2nd-antibody, respectively. The labelled cells were imaged by confocal microscopy. The tiny dots were nonspecific labeling and could be minimized when mouse serum was used to block the binding sites. We thus ignored the tiny dots when examining colocalization of the two. Each high-KCl treated cell presents on average 6-10 bright puncta containing both CHGB and vATPase. The low KCl-treated cells have only 0-2 due to a basal level of granule release. Three puncta showing colocalization of CHGB and vATPase A2 were marked by red circles. These may be locations where multiple granules were released. The resolution limitation did not allow us to recognize individual granules.

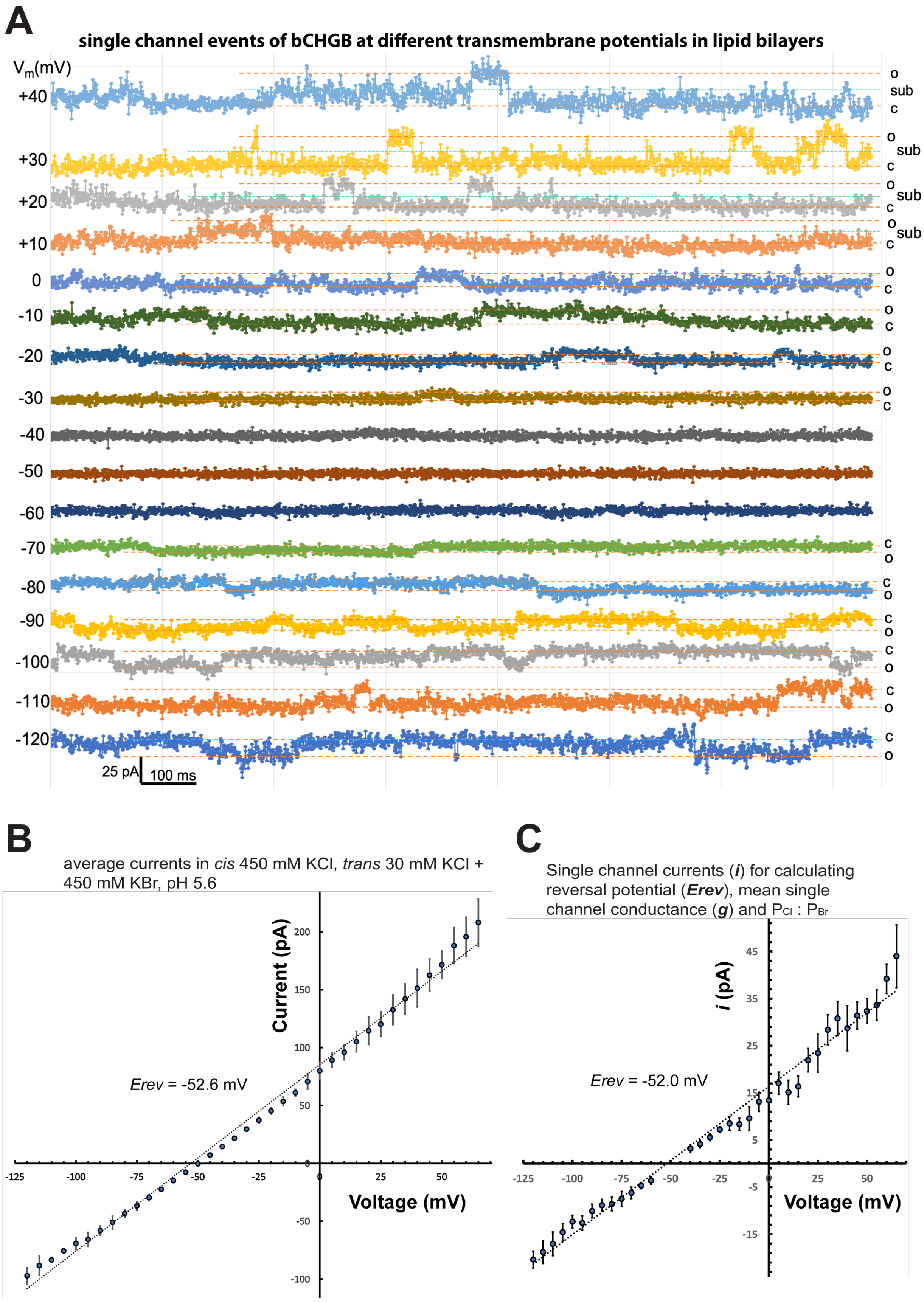
A
single channel events of bCHGB at different transmembrane potentials in lipid bilayers
Vm(mV)
+40
+30
+20
+10
0
-10
-20
-30
-40
-50
-60
-70
-80
-90
-100
-110
-120
o
sub
c
25 pA
100 ms
B
average currents in cis 450 mM KCl, trans 30 mM KCl + 450 mM KBr, pH 5.6
Current (pA)
Voltage (mV)
Erev = -52.6 mV
-125
-100
-75
-50
-25
0
25
50
200
150
100
50
-50
-100
C
Single channel currents (i) for calculating reversal potential (Erev), mean single channel conductance (g) and PCl : PBr
i (pA)
Voltage (mV)
Erev = -52.0 mV
45
35
25
15
5
-5
-15

Supplementary Figure 3. Reversal potentials defined by single channel activities and small macroscopic currents agree well. (A). Single channel events recognizable from currents recorded of bCHGB channels in bilayers at membrane proteins of -120 to 40 mV. Solutions were *cis* 450 mM KCl and *trans* 30 mM KCl + 450 mM KBr with 10 mM MES, pH5.5. Closing (c), opening (o) and sub-conductance (sub) states of the active single channel events were labeled. (**B&C**) Macroscopic currents (**B**) and single channel currents (*I*, **C**)as a function of transmembrane potential (*Vm*) were plotted. Their linear fittings led to reversal potentials of -52.6 and -52.0 mV, respectively. Fitting of the data derived a single channel conductance *g* ~275 pS, and P_{Cl} / P_{Br} ~ 20. Error bars: *s.d.*, n = 6. From all data we have collected, the permeation sequence of the CHGB channel is F^- (1.2) ~ Cl^- (1.0) >> Br^- (~0.05) >>> K^+(~0.01), and its *g* varies with [Cl^-]: ~60 pS (15 mM), ~140 pS (150 mM), and ~ 275 pS (450 mM).

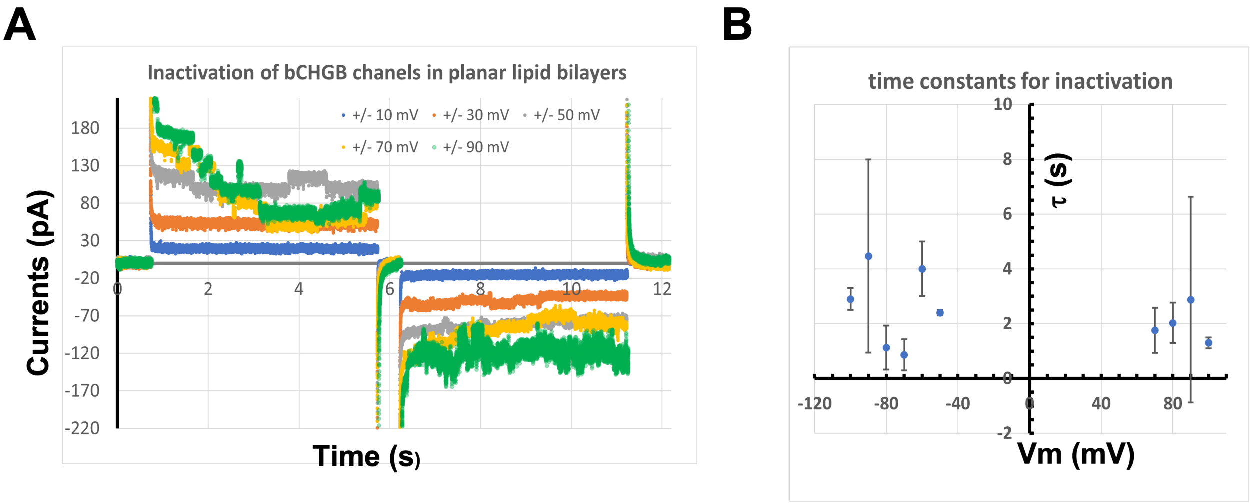

Supplementary Figure 4. Inactivation of bCHGB channels in presence of Mg^{2+}.
(A). Typical small macroscopic currents recordings in solutions of (cis/trans, mM) 150/150 KCl, 2.0 MgCl2, and 5.0 MES-HCl, pH5.6. Longer pulses (5-seconds) were used. **(B).** Single exponential fittings were performed to estimate time constants (τ) for inactivation from 4 different recordings. The average time constants were ~2 seconds in Vm of +70 to +100 mV, and ~1-4 seconds in Vm of -60 to -100 mV.

A

B

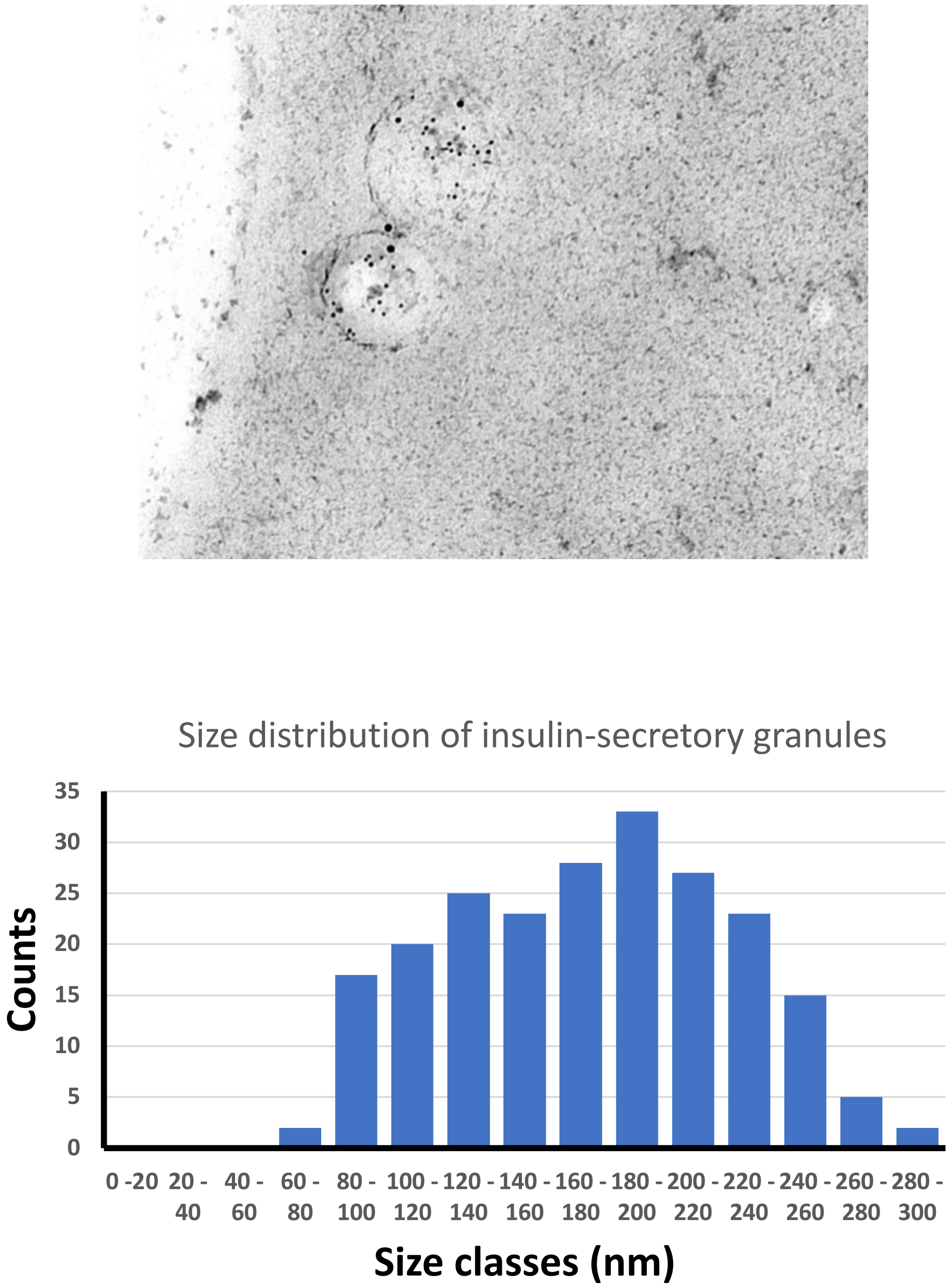

Supplementary Figure 5. Staining specificity and granule size distribution in HPF-immuno-EM. (**A**). A typical image showing specific staining of granules insider cells. Antibodies were diluted to minimize nonspecific staining in other parts of the cell. (**B**). Size distribution of insulin-secretory granules used in the statistical analysis.

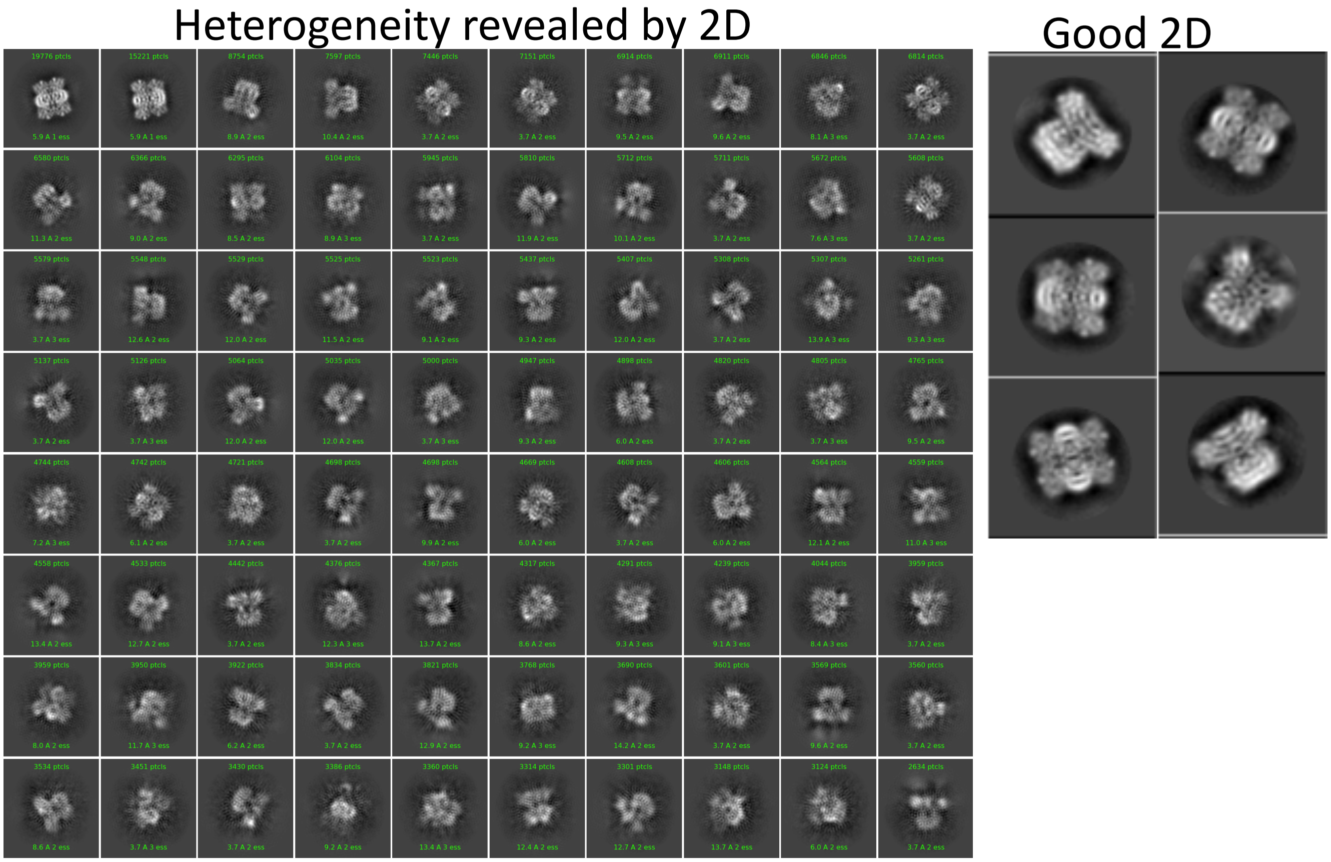

Supplementary Figure 6. Sorting heterogeneous dataset of cryo-EM images of bCHGB dimers.
Left: A montage of 80 2D class averages from 500 generated by 2D classification of individual particle images generated from cryoSPARC.
Right: six selected class averages showing clear separation of the particles from the background, with features suggesting secondary structures of the protein. Particle images in good class averages were then pooled together and used for 3D reconstruction and refinement of the final cryo-EM map.

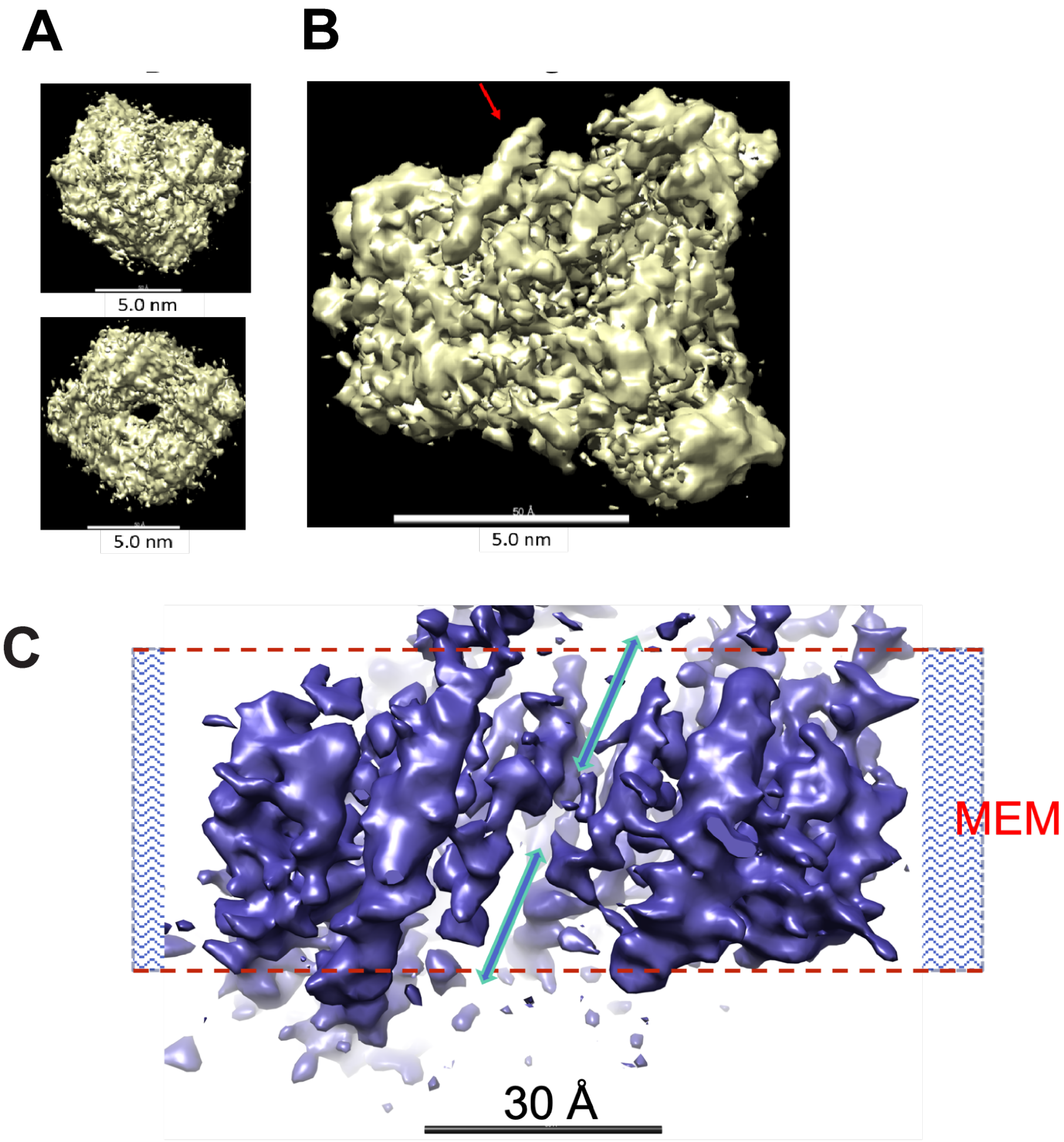

Supplementary Figure 7 A cryo-EM map of CHGB at a nominal 6.8 Å resolution. (**A**). A map from a data set collected in a 200 kV Glacios / Falcon 4 system that was benchmarked at 2.0 Å using apoferritin. Two different views of the 6.8 Å map (FSC=0.143) show the lateral shape (**top**) and one view through the enclosed pore (**bottom**). (**B**). Another lateral view with the C2 axis going from the left to the right shows rod-link densities (e.g. red arrow), probably corresponding to alpha-helices as predicted before [1]. (**C**). A middle section of the map at a higher threshold shows the central cavity sizeable for ion conduction (green arrows). The putative 30 Å hydrophobic core of the membrane is marked out for presentation, and will need further data to validate.

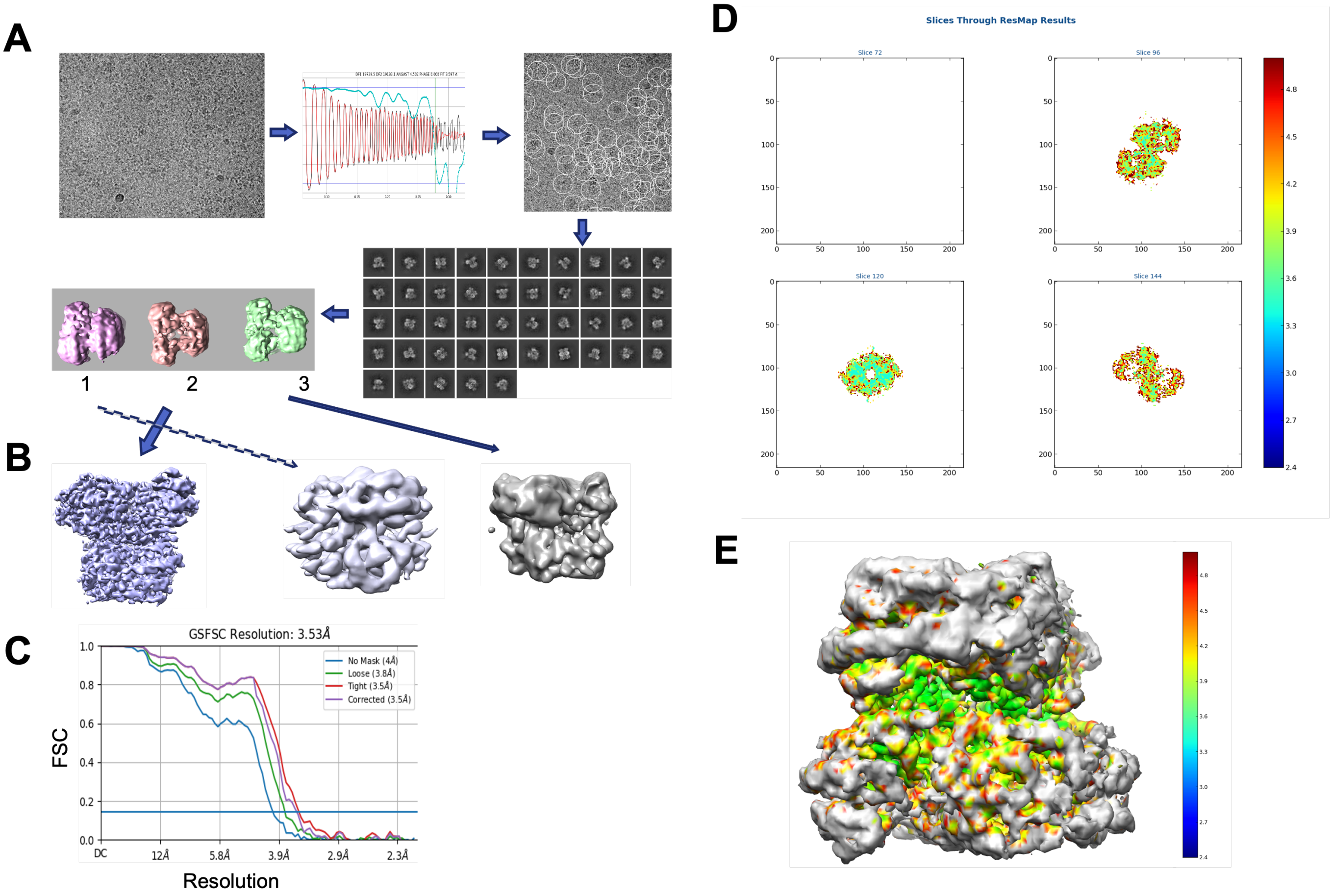

Supplementary Figure 8. Data processing procedure for a large dataset.
(A). Total # of movies: 88,563. Among them, 83,969 were selected based on results of CTF fitting and relative ice thickness. 81,360 entered into particle selection based on the quality of selected particles. The datasets were split into 3 subsets to select the good particles before being merged. After 2 rounds of classification to get rid of images that contained no good particles standing out from the background noise, we were left with about 3.6 million particles. The class averages showed the particle shapes relatively well. After further selection, the dataset was reduced to ~182,900 when only those whose 2D averages show clear structural details were chosen. (B). After *ab initio* reconstruction, ~ 40% of the particles (70,448) were used to calculate the final map of a nominal 3.5 Å resolution. C1 symmetry was used in all steps until C2 was imposed in the last rounds of homogeneous and nonuniform refinements in cryoSPARC. Refinement of the other two reference maps against the leftover particles did not lead to resolutions better than 7.0 Å. (C). FSC for resolution estimation. (D). Map slices showing local resolutions by ResMap. Color bar from 2.4 to 5.0 Å. The slice in the bottom-left panel shows clearly that the core of the map is resolved at ~3.3Å and the periphery is at ~4.8 Å. (E). An unsharpened map showing local resolutions. The final refined maps were deposited at the wwPDB with an EMDB code of EMD-36579.

II. List of reagents

REAGENT or RESOURCE	SOURCE	IDENTIFIER
**Antibodies**		
anti-chromogranin B antibody (Santa Cruz)	Goat	sc-1489
anti-chromogranin B antibody (Santa Cruz)	Rabbit	SC-20135
anti-His antibody (Sigma)	Mouse	27471001 (lot#9535913)
anti-CLC-3 antibody	Rabbit	ab28736 (GR383528-I
anti-chromogranin B	Rabbit	PA1-10839 (TI2638713)
anti-ATP6V0A2	Rabbit	ab96803 (GR9499-19)
**Bacterial and Virus Strains**		
E. coli	available in the lab	K-12 (XL1 blue)
Baculovirus	Invitrogen	Bac-to-Bac expression system
DH10Bac	Invitrogen	Cat #10361012
**Biological Samples**		
Mouse islets	Isolated in the lab	
**Experimental Models: Cell Lines**		
INS-1 832/13 cell line	Wen-Hong Li's lab at UT Southwestern	INS-1
PC-12 cell line	Jerry Shay's lab at UT Southwestern	PC12
**Recombinant DNA**		
pPCDNA3.0-CHGB	constructed in the lab	CHGB
pPcDNA3.0-CHGB ΔMIF	constructed in the lab	CHGBΔMIF
pcDNA3-NPY-ClopHensor	Addgene.com	Plasmid #25939
pcDNA3-ClopHensor	Addgene.com	Plasmid #25938
pfastBac1	Invitrogen	Cat # 10360014
**Software and Algorithms**		
ImageJ	NIH	https://imagej.nih.gov/ij/

III. Supplementary references:

1 Yadav G, Zheng H, Yang Q, Douma LG, Bloom LB, Jiang Q-X. Secretory granule protein chromogranin B (CHGB) forms an anion channel in membrane. *Life Science Alliance* 2018; **1**:e201800139.
